# Author Correction: Nanoscale exchange-bias magnetic tunnel junctions enabled memristive synapse and leaky-integrate-fire neuron for neuromorphic computing

**DOI:** 10.1038/s41467-026-78298-y

**Published:** 2026-09-28

**Authors:** Zanhong Chen, Dehang Zhu, Ao Du, Yuzhang Shi, Wenlong Cai, Zixi Wang, Yuqi Duan, Shiyang Lu, Kaihua Cao, He Zhang, Deming Zhang, Hongxi Liu, Kewen Shi, Weisheng Zhao

**Affiliations:** 1 Fert Beijing Institute, School of Integrated Circuit Science and Engineering, Beihang University, Beijing, China; 2 State Key Laboratory of Spintronics, Hangzhou International Innovation Institute, Beihang University, Hangzhou, China; 3 Truth Memory Tech. Corporation, Beijing, China

**Keywords:** Electronic devices, Electronic devices, Magnetic devices

Correction to: *Nature Communications* https://doi.org/10.1038/s41467-026-70802-8, published online 24 March 2026

In the version of this article initially published, there were inconsistencies in the figures, figure legends and text. In Fig. 1a, dashed lines in layer annotations of the TEM image were oriented incorrectly; in the legend to Fig. 1b, the current text “Magnetoresistance curves” appeared originally as “Magnetic hysteresis loops,” while in the Fig. 1c, d legend “elliptical and circular” appeared originally as “circular and elliptical” and “92.4% and 72.0%” appeared originally as “72.0% and 92.4%.” In Fig. 2h, the unit range now reading top-down as “3 ns, 2 ns, 1.4 ns, 1 ns” was originally interchanged, while in the Fig. 2a caption, “effective voltages” appeared originally as “current amplitudes” and in the Fig. 2c legend, “z direction” appeared originally as “y direction.” In the Fig. 3g legend, “effective voltages” appeared originally as “current densities.”

In the Results section, in the second sentence of the second paragraph, the current text “The magnetoresistance curve” appeared originally as “The hysteresis loop,” while in the text below Eq. (1), in the text now reading “single layer (green) … sandwiched structure (yellow),” yellow and green were originally interchanged. In the Methods “Measurements” section, first paragraph, “Magnetoresistance curves” replaces the original “Magnetic hysteresis loops,” while in the last paragraph of the section, in the text now reading “Lake Shore CRX-VF probe station, capable of applying out-of-plane magnetic fields,” the original “in plane” description has been removed. In the first paragraph of the Methods “Convolutional spiking neural network simulations” section, in the text now reading “τ_pre_ = 2 ns and τ_post_ = 4 ns,” units were originally missing, and in the text now reading “resting potential at *T*_rest_ = 300 K and the firing threshold at *T*_th_ = 450 K,” *T*_rest_ and *T*_th_ appeared originally as “*V*_rest_ and *V*_th_” while the units were missing. The experimental results, data interpretation or the main conclusions of the article are unaffected by the changes. The updates have been made in the HTML and PDF versions of the article.

