# Supplementary material for: Nanoscale exchange-bias magnetic tunnel junctions enabled memristive synapse and leaky-integrate-fire neuron for neuromorphic computing
Source: Nat Commun. 2026 Mar 24;17:4362. doi: 10.1038/s41467-026-70802-8 (PMC13172308; doi:10.1038/s41467-026-70802-8)
Supplement: Supplementary file 1 — Supplementary Information [file 41467_2026_70802_MOESM1_ESM.pdf]

Nanoscale exchange-bias magnetic tunnel junctions enabled memristive synapse and leaky-integrate-fire neuron for neuromorphic computing

Zanhong Chen[1,*], Dehang Zhu[1,*], Ao Du[1,*], Yuzhang Shi[1,*], Wenlong Cai[1,2,#], Zixi Wang[1], Yuqi Duan[1], Shiyang Lu[1], Kaihua Cao[1], He Zhang[1], Deming Zhang[1], Hongxi Liu[3], Kewen Shi[1,2,#], Weisheng Zhao[1,2,#]

[1]Fert Beijing Institute, School of Integrated Circuit Science and Engineering, Beihang University, Beijing 100191, China

[2]State Key Laboratory of Spintronics, Hangzhou International Innovation Institute, Beihang University, Hangzhou 311115, China

[3]Truth Memory Tech. Corporation, Beijing 100086, China

[*]These authors contributed equally: Zanhong Chen, Dehang Zhu, Ao Du, Yuzhang Shi

[#]Corresponding author: Wenlong Cai (caiwenlong1993@buaa.edu.cn)

[#]Corresponding author: Kewen Shi (shikewen@buaa.edu.cn)

[#]Corresponding author: Weisheng Zhao (weisheng.zhao@buaa.edu.cn)

This **Supplementary Information** contains:

Supplementary Note 1. Characterization of the Shape and Fidelity of SOT Pulses.

Supplementary Note 2. Timing diagram of SOT switching and effect of DC read current on TMR.

Supplementary Note 3. Effect of Device Size and Aspect Ratio on SOT Switching.

Supplementary Note 4. Calculation of the Effective Voltage V_{eff}.

Supplementary Note 5. Variation of Multilevel Resistance States in Synaptic Devices with Pulse Width (0.4–5 ns).

Supplementary Note 6. Resistance-State Separation and Its Impact on Network Performance in Synaptic Devices.

Supplementary Note 7. Thermal Stability and Retention Characteristics of Devices.

Supplementary Note 8. Magnetic-Field Robustness of Devices in Different Directions.

Supplementary Note 9. Effect of Antiferromagnetic Domains on Ferromagnetic Domains.

Supplementary Note 10. BCM neuron network implementation

Supplementary Note 11. Neuron and synapse implementation in all-EB-MTJs CSNN.

Supplementary Note 12. Variability and Reproducibility of EB-MTJ Devices.

Supplementary Note 13. Hysteresis loops of intermediate resistance states.

Supplementary Note 14. Temporal Stability of Intermediate Resistance States in Synaptic Devices.

Supplementary Note 15. Confusion Matrix at Best Validation Epoch.

Supplementary Note 16. Trace-based modeling of STDP in EB-MTJ synaptic devices.

Supplementary Fig. 1 Schematic of the measurement setup for pulse waveform characterization.

Supplementary Fig. 2 Morphological characteristics of the 0.4-ns pulse applied to the device.

Supplementary Fig. 3 Schematic of pulse waveforms with different pulse widths.

Supplementary Fig. 4 Schematic of a sequence of ten consecutive 0.4 ns pulses with different time intervals.

Supplementary Fig. 5 Timing diagram of SOT writing pulses and DC read current during SOT-induced switching of device.

Supplementary Fig. 6 Variation of the AP-state resistance of the device under different DC read currents.

Supplementary Fig. 7 Effect of aspect ratio on the number of intermediate states in elliptical devices.

Supplementary Fig. 8 Switching behavior of circular devices with different lateral sizes.

Supplementary Fig. 9 Effect of demagnetization field on switching behavior.

Supplementary Fig. 10 Effect of nucleation-site distribution on switching behavior.

Supplementary Fig. 11 Influence of device size and aspect ratio on SOT-induced switching.

Supplementary Fig. 12 Effective write voltage calculation and bottom electrode structure of the neuron device.

Supplementary Fig. 13 Multilevel resistance characteristics of the device under different pulse widths.

Supplementary Fig. 14 Schematic illustration of the resistance states and error bars as a function of SOT pulse voltage.

Supplementary Fig. 15 Gaussian distributions of different intermediate states of the normalized device resistance.

Supplementary Fig. 16 Gaussian distributions of different intermediate states of normalized resistance for multiple devices.

Supplementary Fig. 17 Effect of different numbers of synaptic resistance states on network accuracy.

Supplementary Fig. 18 Critical switching voltage as a function of pulse width, with fitting in the long-pulse-width regime using a thermal activation model.

Supplementary Fig. 19 Device retention at high temperature.

Supplementary Fig. 20 Magnetoresistance characterization of the device.

Supplementary Fig. 21 Influence of antiferromagnetic domains on ferromagnetic domain morphology.

Supplementary Fig. 22 Triplet STDP fitting with the parameter τ_x, τ_y, A_{3+} and A_{3-}.

Supplementary Fig. 23 Typical triplet-pulse diagram with "pre–post–pre" and "post–pre–post" sequences.

Supplementary Fig. 24 Triplet-STDP-based BCM learning rules.

Supplementary Fig. 25 The comparison of test accuracy of STDP and BCM.

Supplementary Fig. 26 Comparison between real weights and quantized weights in our neuron network.

Supplementary Fig. 27 The test accuracy of different numbers of states and QAT controlled parameter α variation.

Supplementary Fig. 28 STDP rule of long-term plasticity demonstrated for paired spikes.

Supplementary Fig. 29 Distribution statistics of P-state resistance and TMR across multiple devices.

Supplementary Fig. 30 Distribution of SOT critical switching voltages and corresponding switching curves for multiple devices.

Supplementary Fig. 31 Distribution of the number of resistance states across multiple devices.

Supplementary Fig. 32 Device characteristics from foundry fabrication.

Supplementary Fig. 33 Classification accuracy as a function of the write error associated with each synaptic resistance state.

Supplementary Fig. 34 Schematic *R–H* curves of the device at different resistance states.

Supplementary Fig. 35 Time evolution of different intermediate resistance states in the same device.

Supplementary Fig. 36 Confusion Matrix at Best Validation Epoch.

Supplementary Fig. 37 Trace-based modeling of STDP in EB-MTJ synaptic devices.

Supplementary Table 1 Parameters extracted from paired-STDP and triplet-STDP, optimized using the minimal triplet model.

Supplementary Table 2 Comparison and benchmark among different typical multi-state synapse devices.

Supplementary Table 3 Comparison and benchmark among different typical spiking neuron devices.

Supplementary Note 1. Characterization of the Shape and Fidelity of SOT Pulses

In this work, two types of programming signals are employed: single SOT pulses with pulse widths as short as 0.4 ns, and pulse trains consisting of up to ten consecutive pulses with variable inter-pulse intervals. To investigate the signal quality and amplitude of these two types of pulses, we present the experimentally measured waveforms of both signal types through the setup shown in Supplementary Fig. 1.

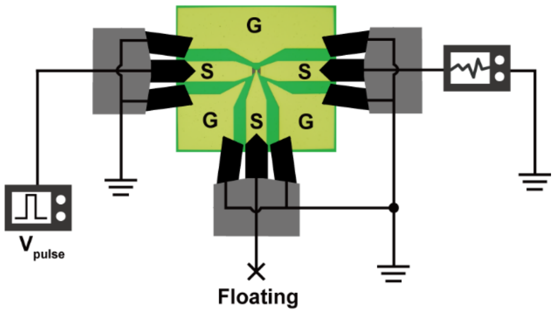

Supplementary Fig. 1 Schematic of the measurement setup for pulse waveform characterization.

A Keysight M8190A arbitrary waveform generator (maximum sampling rate of 8 GHz) was used to generate the pulses, which were amplified and then applied to the bottom electrode of the device. The device shown in the center of the setup is the GSG-compatible structure used in this work. One side of the device was connected to the waveform generator via a GSG probe, while the other side was connected to a high-resolution oscilloscope through another GSG probe for signal readout.

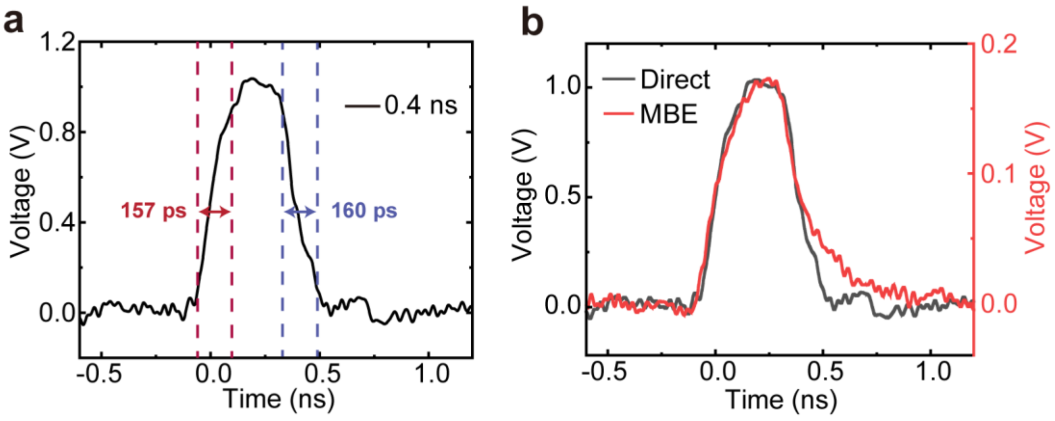

Supplementary Fig. 2 Morphological characteristics of the 0.4-ns pulse applied to the device. **a** A 0.4-ns SOT pulse with an amplified amplitude of 1 V. The rise and fall times are approximately 160 ps. **b** Comparison of the 0.4-ns pulse measured at the input and after transmission through the metal bottom electrode (MBE). After passing through the bottom electrode, the rise time shows no noticeable change, while the fall time increases from 160 ps to 300 ps.

Supplementary Fig. 2a shows a representative 0.4 ns SOT pulse generated by an arbitrary waveform generator, with a nominal amplitude of 1 V after amplification. The measured waveform demonstrates that the pulse maintains a well-defined shape at the sub-nanosecond timescale, with a rise time of approximately 157 ps, a fall time of approximately 160 ps, both defined using the 10%–90% voltage criterion, and an effective pulse width of about 407 ps. The measured pulse amplitude is ~1.007 V, in excellent agreement with the nominal post-amplification value, confirming the high fidelity and accuracy of the applied SOT pulses. The red curve in Supplementary Fig. 2b shows the pulse waveform measured after transmission through the device bottom electrode. Compared with the directly measured waveform, the rising edge remains nearly unchanged, whereas the falling edge is slightly extended. Nevertheless, the overall pulse width shows no significant variation. This can be understood as a result of the effective RC effect introduced by the bottom electrode[4,5]. While the leading edge is dominated by the initial fast excitation and remains largely unaffected, the high-frequency components contributing to the trailing edge are partially attenuated, resulting in a prolonged decay. As a consequence, the pulse shape becomes slightly asymmetric, while the full pulse width remains nearly unchanged and does not affect the switching behavior of the device.

Since both the output impedance of the arbitrary waveform generator and the input impedance of the oscilloscope are 50 Ω, while the resistance of the device bottom electrode is 382.8 Ω, the expected voltage division yields V_{direct} = 50/(50+50)*V_{input} = 0.5V_{input} and V_{MBE} = 50/(50+50+382.8)*V_{input} = 0.1035V_{input}, such that V_{MBE} = 0.207V_{direct}. Experimentally, the measured voltages are 1.007 V and 0.171 V, respectively, corresponding to $V_{\text{MBE,real}}$ = 0.170$V_{\text{direct,real}}$. The deviation between the expected and measured values is approximately 17.9%, which we attribute mainly to impedance mismatch between the device bottom electrode and the 50 Ω measurement system. Nevertheless, under ultrashort pulse conditions (0.4 ns), this level of attenuation is within an acceptable range.

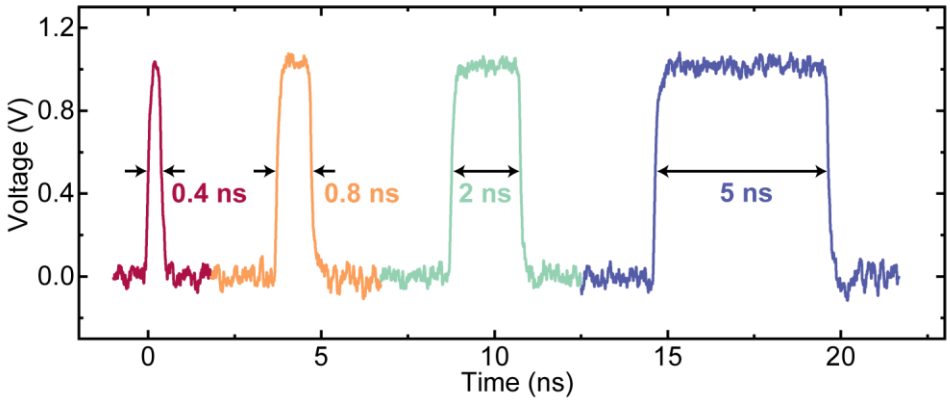

Supplementary Fig. 3 Schematic of pulse waveforms with different pulse widths.

We further examine the waveform quality for single SOT pulses with different pulse widths. As shown in Supplementary Fig. 3, pulses with widths of 0.4, 0.8, 2, and 5 ns all exhibit stable and well-defined waveforms, indicating that the single-pulse signals used for device switching are robust over the investigated timescale range.

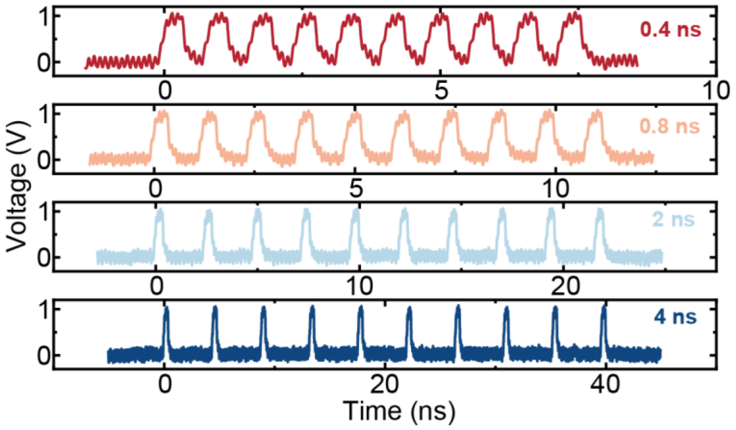

Supplementary Fig. 4 Schematic of a sequence of ten consecutive 0.4 ns pulses with different time intervals.

In addition, we evaluate the fidelity of the pulse sequences employed to implement the leaky-integrate-and-fire (LIF) neuron functionality. Supplementary Fig. 4 shows the measured waveforms of pulse trains consisting of ten consecutive 0.4 ns SOT pulses with different inter-pulse intervals. The pulse trains retain high signal fidelity across all tested conditions – even at an extremely short interval of 0.4 ns, the pulse amplitude and temporal profile remain comparable to those of an isolated single pulse, without observable distortion induced by pulse crowding. These results collectively confirm the accuracy and reliability of the pulse waveforms used to realize the LIF neuron operation.

Supplementary Note 2. Timing diagram of SOT switching and effect of DC read current on TMR

The measurement protocol in Fig. 2a of the main text follows a well-defined write–read sequence, which is illustrated in Supplementary Fig. 5. Specifically, a single write pulse with a duration of 0.4 ns is first applied to the device. After the write operation is completed, a waiting time of approximately 300 ms is introduced before the resistance is read. The resistance is then measured using the small sensing current of 1 μA for a duration of about 500 ms. After an additional interval of approximately 1 s, the next write pulse is applied, and the same write–read sequence is repeated. As a result, the intermediate resistance levels observed in Fig. 2a correspond to retained and well-defined device states established during the write operation, rather than to transient changes during readout.

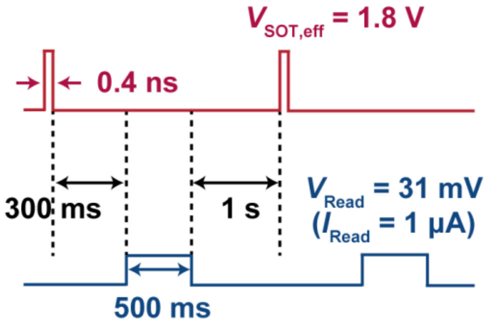

Supplementary Fig. 5 Timing diagram of SOT writing pulses and DC read current during SOT-induced switching of device.

Given this measurement sequence, the write process has fully terminated well before the readout begins, and the temperature rise induced by the 0.4 ns write pulse is expected to relax back to a level close to room temperature within the 300 ms interval, such that no appreciable thermal accumulation remains during the read operation. Importantly, all resistance states are read using the same small sensing current of 1 μA. At this read current, both the temperature increase due to Joule heating and the effect of the read voltage on the TMR are minimal. Supplementary Fig. 6 shows the dependence of the device resistance on the applied DC read current. From the fitting, we estimate that, compared with the resistance measured without current injection, the AP-state resistance decreases by only ~1.02% when a read current of 1 μA (corresponding to a read voltage of 31.3 mV) is applied, corresponding to a TMR variation of less than 0.9%. Therefore, the DC read current of 1 μA employed in our measurements has a negligible impact on the resistance states of the device.

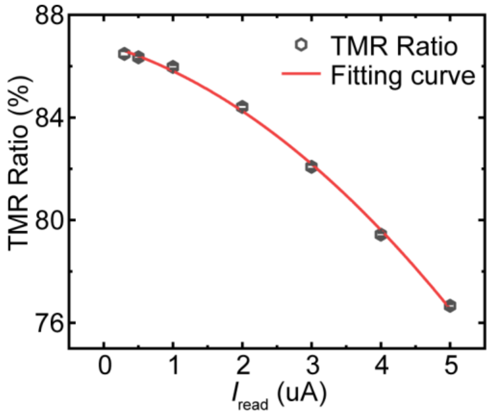

Supplementary Fig. 6 Variation of the AP-state resistance of the device under different DC read currents.

Supplementary Note 3. Effect of Device Size and Aspect Ratio on SOT Switching

To clarify the key factors governing the distinct switching behaviors observed in our devices, we have carried out additional micromagnetic simulations and carefully compared them with the experimental results, which allows us to further clarify the physical origin. The distinct switching behaviors observed in elliptical and circular devices, namely progressive (analog-like) versus abrupt (binary-like) reversal, respectively, are not determined by the device geometry alone. The aspect ratio, device size, demagnetization field, and domain wall can properly influence the device's properties.

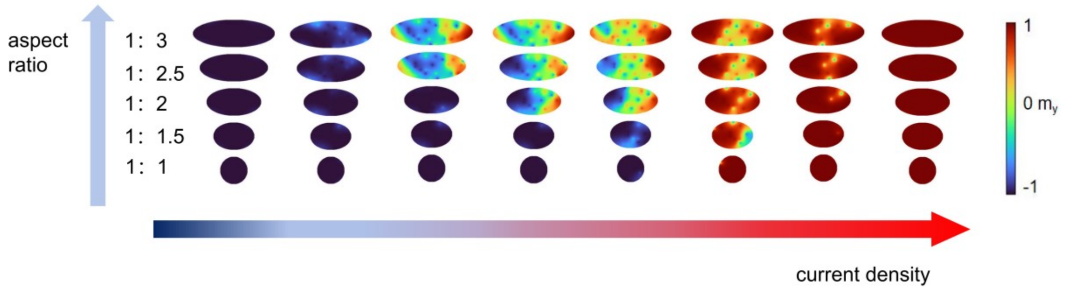

Supplementary Fig. 7 Effect of aspect ratio on the number of intermediate states in elliptical devices.

First, we focused on the geometric parameters, and found that the aspect ratio plays the most critical role in enabling multistate behavior. As shown in Supplementary Fig. 7, increasing the aspect ratio leads to a pronounced increase in the number of stable intermediate states, a trend that is consistently reproduced under different material conditions investigated in this study. The underlying physical origin of the aspect-ratio–dependent multistate behavior can be more clearly understood by considering the dynamic switching process. During the application of the writing current, the combination of the spin–orbit torque and Joule heating leads to a partial decoupling of antiferromagnetic grains from the ferromagnetic layer. As a result, the local exchange bias and the associated pinning are temporarily suppressed, allowing the corresponding regions of the ferromagnet to reverse more readily under the Oersted field. This process gives rise to intermediate multi-domain configurations in the ferromagnetic layer. After releasing the writing current, the stability of these intermediate configurations becomes strongly dependent on the device's aspect ratio. In elliptical devices with a higher aspect ratio, domain walls preferentially distribute along the short axis, resulting in a shorter domain-wall length and a lower total domain wall energy. These low-energy multi-domain states can remain stable for a sufficiently long time, enabling the previously decoupled antiferromagnetic grains to re-establish exchange coupling with the ferromagnet. This re-coupling process generates a newly oriented exchange-bias pinning field, which effectively locks the corresponding ferromagnetic domains into stable configurations. Consequently, a larger number of robust and distinguishable resistance states can be formed and retained over long timescales.

In addition to the aspect ratio, the lateral size of the device also influences whether the magnetization reversal proceeds in a binary-like or progressive manner, as shown in Supplementary Fig. 8. When the device size is sufficiently small (typically below ~50 nm), the ferromagnetic layer tends to remain in a single-domain state. In this regime, even under SOT-induced heating and partial suppression of exchange bias, the formation of stable multidomain configurations is energetically unfavorable, leading to predominantly abrupt, binary switching behavior. As the lateral size increases, the ferromagnetic layer can support localized or multi-domain reversal during the application of the writing current, and intermediate resistance states appear in circular devices. However, owing to the circular geometry, the associated domain walls are typically long, resulting in a relatively high domain-wall energy. After the writing current is removed, these high-energy multidomain configurations are unstable and tend to collapse back to the initial state on a short timescale, often before the antiferromagnetic grains can fully re-establish exchange coupling with the ferromagnet. Consequently, only a limited number of

intermediate states can be retained in large circular devices. In contrast, high–aspect-ratio elliptical devices accommodate domain walls with significantly shorter lengths, thereby reducing the domain-wall energy of the intermediate configurations. This allows the multidomain states to remain stable for a sufficiently long time after the end of the writing pulse, enabling the reformation of exchange bias and the establishment of robust pinning. As a result, while increasing the device size promotes the emergence of multidomain or localized reversal, the ability to realize a large number of stable and well-separated resistance states is primarily governed by whether the device geometry permits these configurations to be stabilized with a low domain-wall energy penalty, highlighting the dominant role of the aspect ratio.

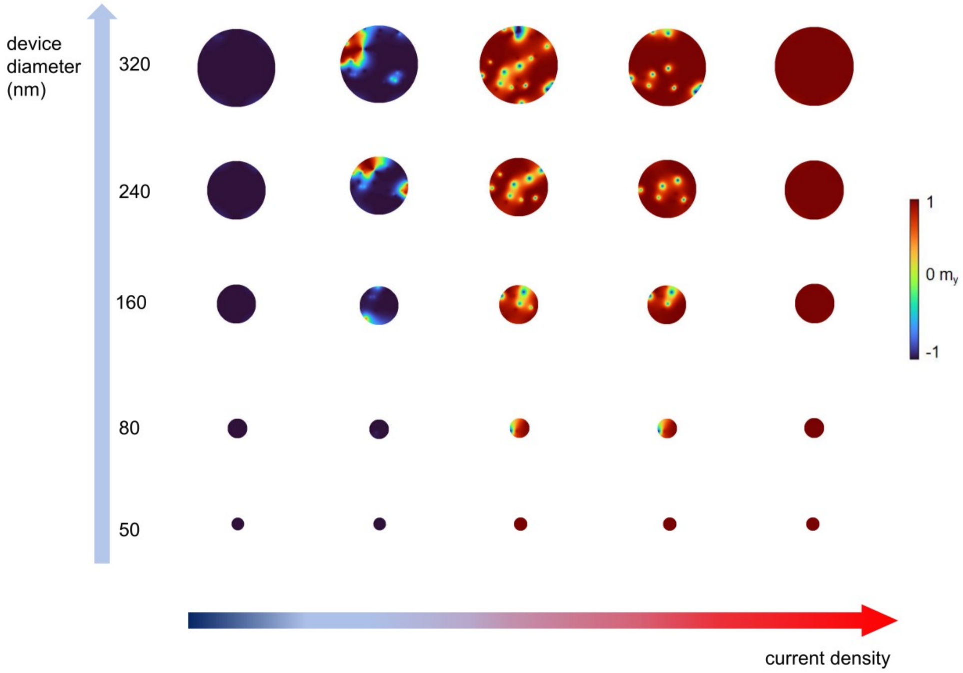

Supplementary Fig. 8 Switching behavior of circular devices with different lateral sizes.

We further systematically examined other magnetic factors that may influence the switching behavior. Regarding the demagnetization field, we tuned the demagnetization energy term in micromagnetic simulations by varying the free-layer thickness and compared thicknesses of 1.8 nm and 2.0 nm (Supplementary Fig. 9). For free-layer thicknesses close to the experimentally used value of 1.9 nm, variations in thickness have only a minor effect on the switching behavior. In this regime, thickness mainly modifies the overall energy barrier, while the switching pathway, the number of accessible intermediate states, and their dependence on the aspect ratio and the device size remain essentially unchanged. This demonstrates that, around 1.9 nm, the free-layer thickness does not play a dominant role in determining whether the device exhibits progressive multistate behavior or abrupt binary switching.

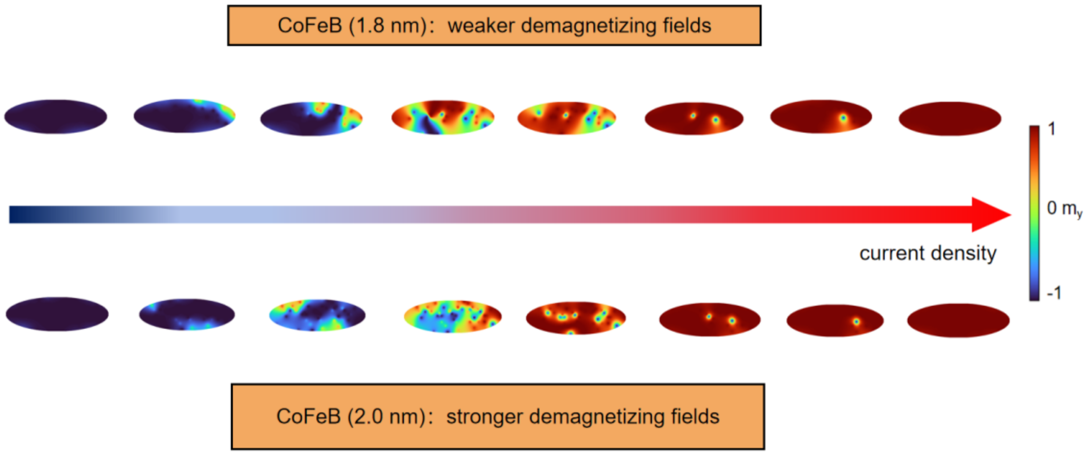

Supplementary Fig. 9 Effect of demagnetization field on switching behavior.

Meanwhile, we also investigated the influence of domain-wall nucleation-site distribution on the switching characteristics (Supplementary Fig. 10). By statistically comparing different initial nucleation positions at the device edges and in the interior, we find that the nucleation sites primarily affect the spatial unfolding of the reversal process and the sequence of local switching events, but do not alter the number of stable intermediate states nor their overall dependence on the aspect ratio in a statistical sense. In other words, the nucleation-site distribution determines where the reversal initiates, but not whether multistate behavior can be formed, and therefore does not constitute the fundamental origin of the observed multilevel resistance states. Moreover, as for the factor of SOT efficiency, the spin–orbit torque is mainly generated by the spin Hall effect in the bottom Pt layer. Since the material system and injection scheme are identical for different geometries, both the efficiency and direction of the spin–orbit torque remain essentially the same across devices, and thus are not responsible for the observed differences between progressive and abrupt switching behaviors.

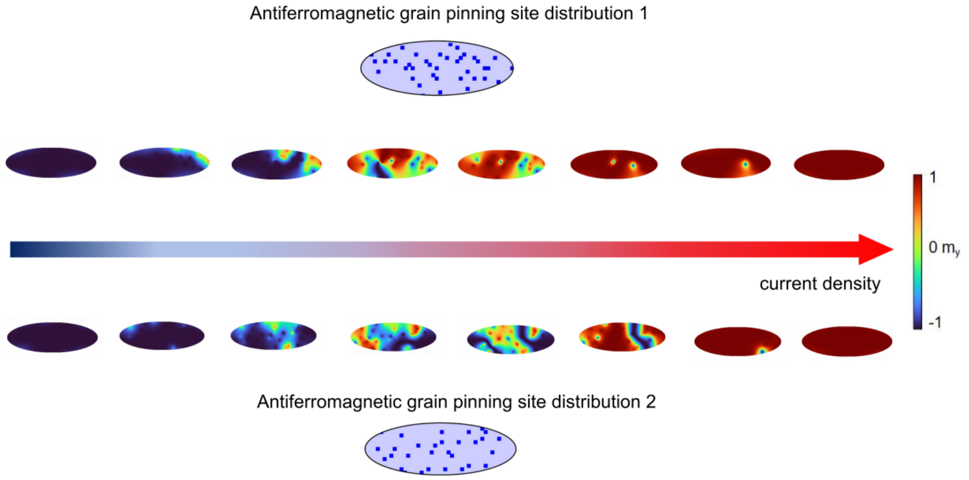

Supplementary Fig. 10 Effect of nucleation-site distribution on switching behavior.

Finally, compared with the simulation results, we also conducted experimental tests on the different devices. Supplementary Fig. 11(a–c) presents representative writing loops of circular

EB-MTJs measured across a wide range of lateral sizes under 1-ns SOT pulses. The data consistently show that, as the device size increases, intermediate resistance states progressively emerge, indicating a reproducible transition from nearly coherent to multidomain or localized reversal during the writing process. Furthermore, when the device geometry is systematically modified to increase the aspect ratio, as shown in Supplementary Fig. 11(d–i), the write windows associated with different resistance states become wider and more clearly separated, and a larger number of distinct multistate levels can be reliably accessed. These trends are observed reproducibly across devices with different sizes and aspect ratios, confirming that the geometry-dependent switching behavior is robust rather than device-specific. In agreement with the simulation analysis, the experimental results also demonstrate that the multistate capability of the present system is governed by a combination of the aspect ratio and lateral size. The aspect ratio is particularly effective in stabilizing multiple intermediate states, whereas the lateral size plays a crucial role in determining whether multidomain or nearly coherent switching occurs, especially when the device dimensions are reduced down to tens-of-nanometers regime. Based on this physical picture, we employ high–aspect-ratio elliptical devices to obtain rich and stable multistate behavior for synaptic units, while small circular devices are selected to realize nearly coherent binary switching for neuronal units, thereby establishing a clear and robust functional separation at the device level.

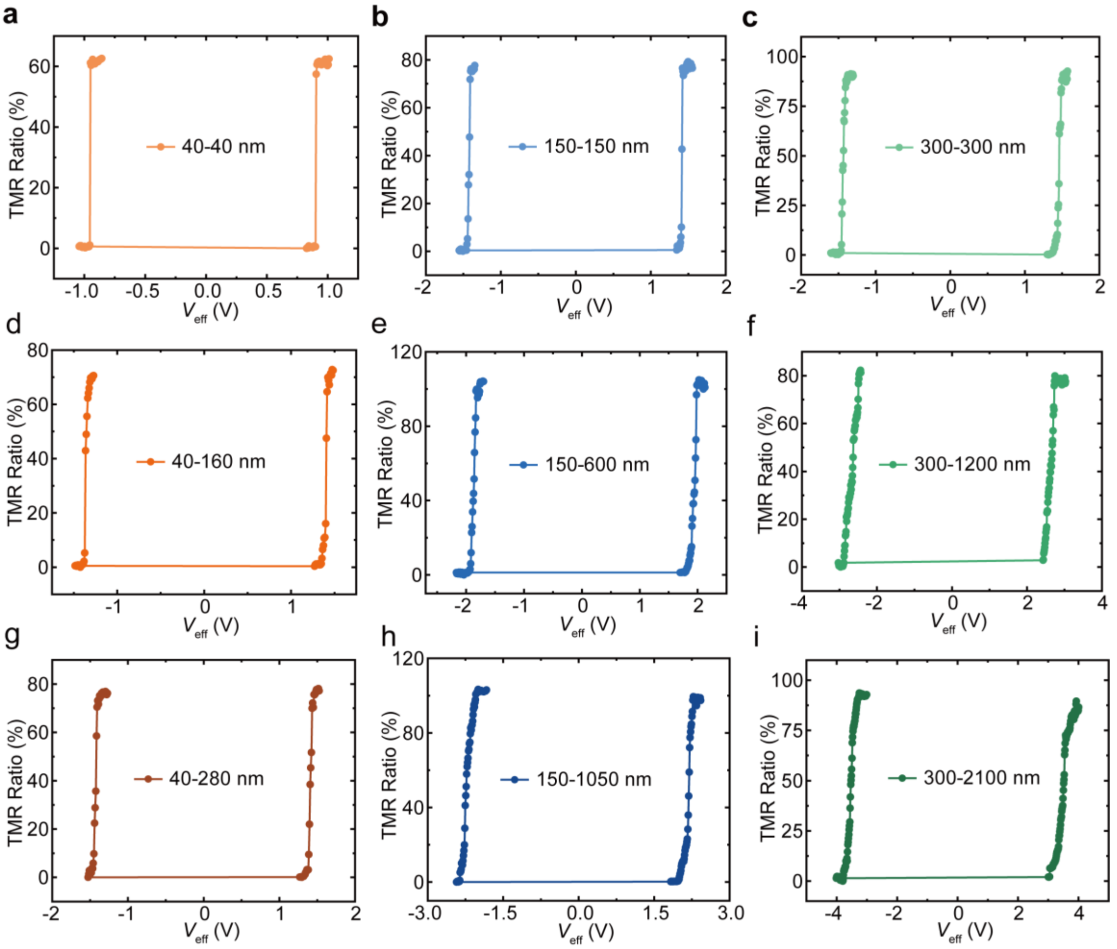

Supplementary Fig. 11 Influence of device size and aspect ratio on SOT-induced switching. a–c Switching loops of circular devices with varying diameters under a 1-ns SOT pulse. As the device size increases, a slight increase in the number of intermediate resistance states is observed

during switching. **d–i** Switching traces of elliptical devices with different sizes and aspect ratios under a 1-ns SOT pulse. A pronounced increase in the number of intermediate resistance states is observed with increasing device size and aspect ratio.

Supplementary Note 4. Calculation of the Effective Voltage V_{eff}

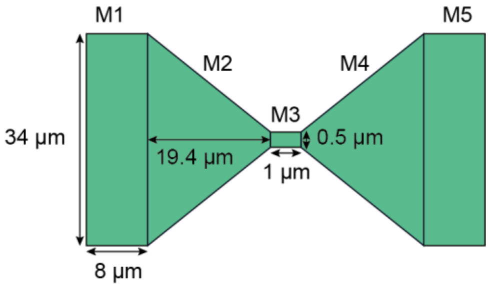

Supplementary Fig. 12 Effective write voltage calculation and bottom electrode structure of the neuron device. The structure and dimensional parameters of the bottom electrode in the neuron device.

As shown in Supplementary Fig. 12, the Pt bottom electrode of our device can be divided into five segments (M1-M5). M1 and M5, as well as M2 and M4, are symmetric rectangular and isosceles trapezoidal regions, respectively, while M3 is a rectangular segment directly beneath the MTJ. Therefore, we consider the voltage drop across M3 as the effective write voltage during device operation. The total resistance of the bottom electrode is measured to be $R_{\mathrm{MBE}} = 2R_{\mathrm{M1}} + 2R_{\mathrm{M2}} + R_{\mathrm{M3}}$. The resistance of each trapezoidal region (e.g., M2) can be expressed as:

$$R_{\mathrm{M2}} = \rho \cdot \frac{L}{t \cdot W_{\mathrm{eff}}} \tag{1}$$

where ρ is the resistivity of Pt, L is the length of the trapezoid, t is the electrode thickness, and $W_{\mathrm{eff}} = \frac{W_{\max}+W_{\min}}{2}$ is the equivalent width of M2. Therefore, the number of square resistances for M2 is:

$$N_{\mathrm{M2}} = \frac{L}{W_{\mathrm{eff}}} = \frac{19.4}{\frac{34+0.5}{2}} \approx 1.12464. \tag{2}$$

Summing over all segments, the total number of squares in the bottom electrode is:

$$N = 2N_{\mathrm{M1}} + 2N_{\mathrm{M2}} + N_{\mathrm{M3}} = \frac{2\times8}{34} + \frac{2\times2\times19.4}{34.5} + \frac{1}{0.5} \approx 4.71986. \tag{3}$$

Accordingly, the voltage drop across M3 can be calculated as:

$$V_{\mathrm{eff}} = V_{\mathrm{MBE}} \cdot \frac{N_{\mathrm{M3}}}{N} \approx 0.427 V_{\mathrm{MBE}}. \tag{4}$$

Given the measured total resistance of the bottom $R_{\mathrm{MBE}} = 368.9\ \Omega$, the resistance of M3 $R_{\mathrm{M3}} \approx 157.5\ \Omega$. Thus, the resistivity of the Pt bottom electrode can be extracted from the known dimensions and calculated square resistance:

$$\rho = R_{\mathrm{M3}} \cdot \frac{t \cdot W_{\mathrm{eff}}}{L} \approx 62.5\ \mu\Omega \cdot \mathrm{cm}. \tag{5}$$

Supplementary Note 5. Variation of Multilevel Resistance States in Synaptic Devices with Pulse Width (0.4–5 ns)

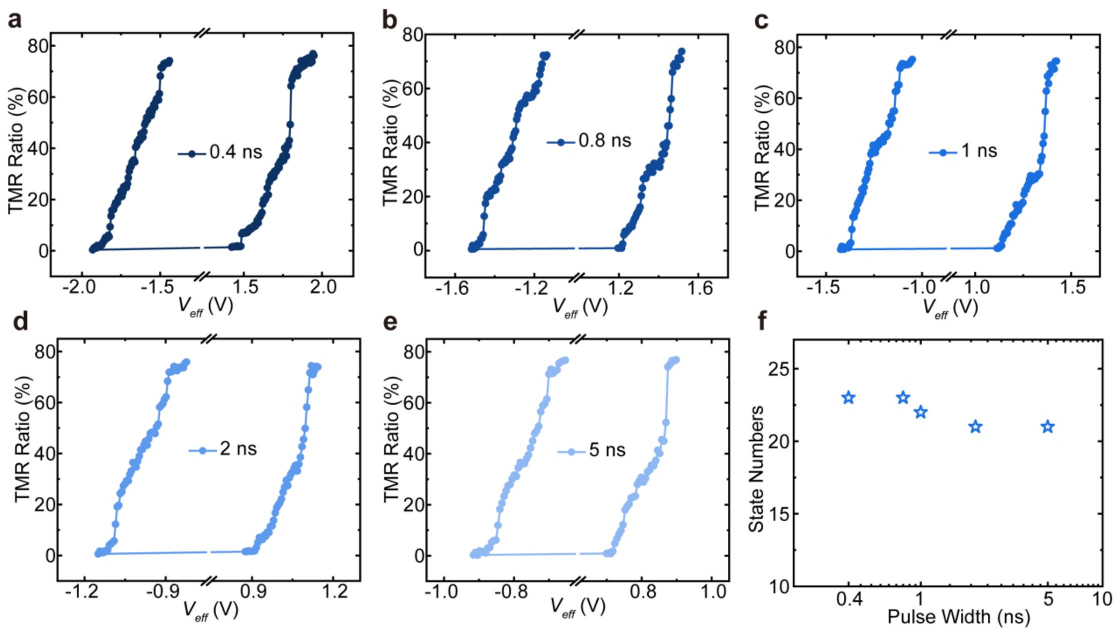

Supplementary Fig. 13 Multilevel resistance characteristics of the device under different pulse widths. a–e R–V switching curves of the device under SOT pulses with widths of 0.4–5 ns. **f** Statistics of the number of resistance states at different pulse widths.

We have supplemented the SOT switching characteristics of the elliptical synaptic devices for pulse widths ranging from 0.4 to 5 ns, as shown in Supplementary Fig. 13(a-e). Clear multilevel resistance behavior is observed across this entire pulse-width range. Taking a tunneling magnetoresistance (TMR) difference greater than 2% as the criterion for distinguishing resistance states, we counted the number of accessible resistance levels at different pulse widths and summarized the results in Supplementary Fig. 13f.

Although the number of resistance states decreases with increasing pulse width, more than 20 distinct resistance states are still achieved for all pulse widths between 0.4 and 5 ns, indicating robust multilevel characteristics even over a wide pulse-width range. Notably, for the 0.4-ns pulses, the full multilevel writing window spans from 1.38 to 1.94 V, corresponding to approximately 33.5% of the critical switching threshold defined at $(R_{AP}+R_P)/2$. This relatively wide programming margin enables reliable and fine control of the device resistance during ultrafast programming.

Supplementary Note 6. Resistance-State Separation and Its Impact on Network Performance in Synaptic Devices

In our manuscript, Fig. 2a is intended to demonstrate the maximum number of resistance states achievable in a single device under single-shot programming, serving as a characterization of intrinsic device capability rather than the effective number of reliably distinguishable states. To quantitatively evaluate the separability of different resistance states, we performed repeated reset–set measurements on multiple devices using 100 cycles of 0.4-ns SOT pulses. For each

programming voltage, we extracted the mean value and standard deviation of the normalized resistance states. Supplementary Fig. 14 shows a representative example of the normalized resistance states and their corresponding standard deviations as a function of the applied voltage.

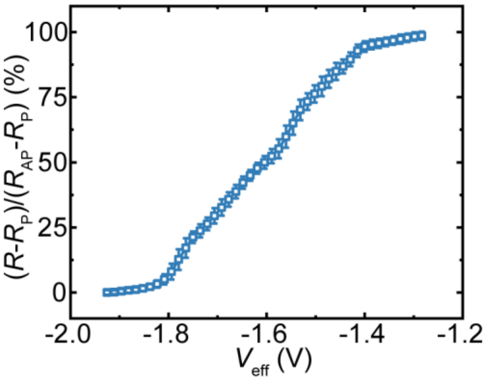

Supplementary Fig. 14 Schematic illustration of the resistance states and error bars as a function of SOT pulse voltage. Error bars representing the standard deviation (SD).

Following the representation used in Fig. 7 of Agrawal et al.[6], we selected a subset of resistance states along the switching curve that exhibit relatively good separation and modeled their resistance distributions using Gaussian functions:

$$f(x) = \exp[-\frac{(x-\mu)^2}{2\sigma^2}] \quad (6)$$

where μ denotes the mean value of the normalized resistance state and σ represents the standard deviation. The resulting resistance distributions are shown in Supplementary Fig. 15.

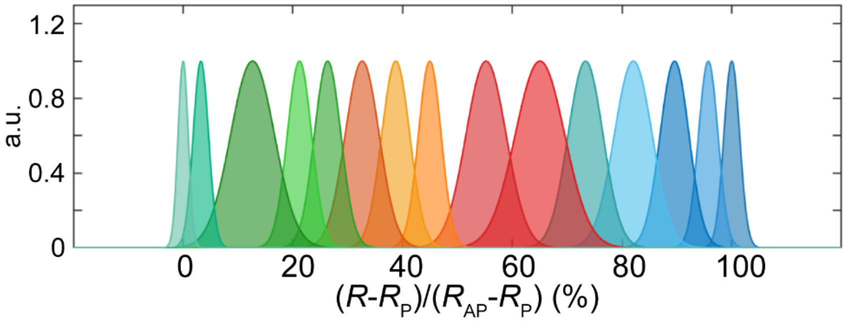

Supplementary Fig. 15 Gaussian distributions of different intermediate states of the normalized device resistance.

As correctly pointed out by the reviewer, due to the limited switching ratio and the presence of write variability, the number of effectively distinguishable resistance states is smaller than 25, and is approximately 15 in practice. To provide a more comprehensive picture, we repeated the same analysis on ten different devices. The statistical distribution of distinguishable resistance states is summarized in Supplementary Fig. 16, showing that the number of separable states lies in the range of 11–15 across these devices.

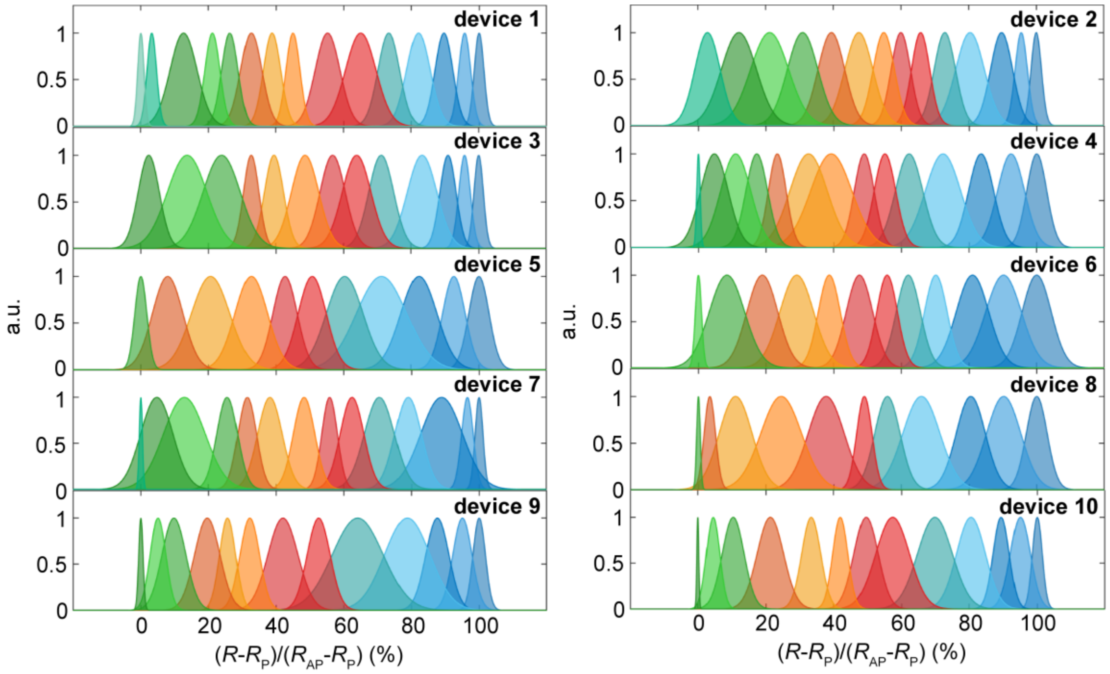

Supplementary Fig. 16 Gaussian distributions of different intermediate states of normalized resistance for multiple devices.

To assess the effect of a reduced number of synaptic resistance states on network performance, we first decreased the number of states in the full EB-MTJ CSNN from 25 (as in the main text) to 15 and then simulated the corresponding network behavior, as shown in Supplementary Fig. 17. The results show that the network with 15 states exhibits only a marginal reduction in maximum accuracy, from 96.2% to 94.8%, throughout the training process. Overall, this demonstrates that the network is largely robust to variations in the number of synaptic states, indicating that the current synaptic devices are sufficient to support high-precision gesture recognition.

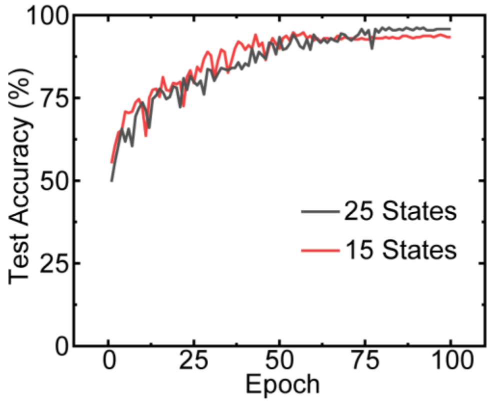

Supplementary Fig. 17 Effect of different numbers of synaptic resistance states on network accuracy

Although this slight reduction in state number has minimal impact on performance, future work will focus on improving the separation between resistance states to increase the number of effectively distinguishable states.

Supplementary Note 7. Thermal Stability and Retention Characteristics of Devices

To demonstrate the thermal stability of our devices, we employed two complementary approaches: (i) estimation of the thermal stability factor based on a theoretical model, and (ii) a thermally accelerated aging experiment at elevated temperature.

First, we estimate the retention based on an ideal single-domain magnetic model. For the circular neuron devices, the ferromagnetic layer exhibits two lowest-energy stable states, namely the AP and P states (for the synaptic devices, the midpoint of the TMR can be taken as the boundary). These two stable states are separated by an energy barrier E_b. Assuming only thermally induced single-domain magnetization reversal, the average switching time τ between the two states follows the Néel–Brown law:

$$\tau = \tau_0 \exp(\frac{E_b}{k_B T}) \quad (7)$$

where τ_0 is the attempt time (typically 10^{-9} s), k_B is the Boltzmann constant, and T is the temperature. The quantity $\Delta = E_b/k_B T$ is commonly defined as the thermal stability factor.

Next, for conventional SOT-driven MTJ switching, the switching behavior can be divided into two regimes depending on the pulse width, as reported in previous studies[2,10,11]. As illustrated in Supplementary Fig. 18, when the pulse width is short, the switching occurs in a non-fully thermally activated regime, where the critical switching voltage shows an approximately inverse dependence on pulse width. In contrast, for longer pulse widths, switching occurs in the thermally activated regime, where the critical switching voltage exhibits a linear dependence on the pulse width. In this regime, the relationship between the critical switching voltage V_C and the pulse width τ can be expressed as

$$V_C = V_{C0}[1 - \frac{k_B T}{E_b} \ln(\frac{\tau}{\tau_0})] \quad (8)$$

where V_{C0} is the critical switching voltage in the absence of thermal activation. By fitting this relation, an estimate of the thermal stability factor can be obtained, which in turn allows the retention time to be inferred. To ensure that the switching process is dominated by thermal activation, we performed the fitting using critical switching voltages corresponding to pulse widths longer than 10 μs. From this analysis, the extracted thermal stability factor is approximately 113.3, which is well above the typical requirement for ten-year data retention at room temperature.

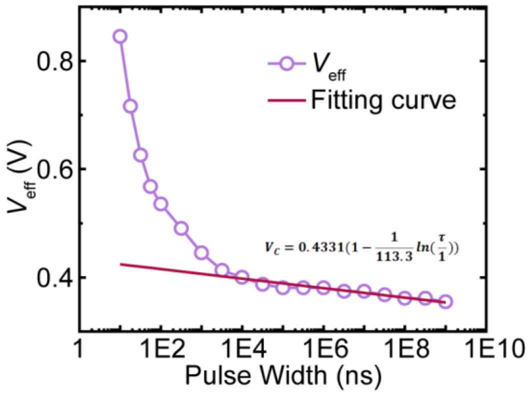

Supplementary Fig. 18 Critical switching voltage as a function of pulse width, with fitting in the long-pulse-width regime using a thermal activation model.

Moreover, to complement the analytical estimation, we evaluated the retention using a thermally accelerated aging test. Supplementary Fig. 19a shows the switching behavior of the device at an elevated temperature of 470 K using 100-ns SOT pulses. Then, the device was programmed into an intermediate resistance state under these conditions, and the resistance was monitored continuously. As shown in Supplementary Fig. 19b, the resistance state remains stable for more than 2650 minutes (>44 hours) at 470 K, indicating good thermal robustness of the device.

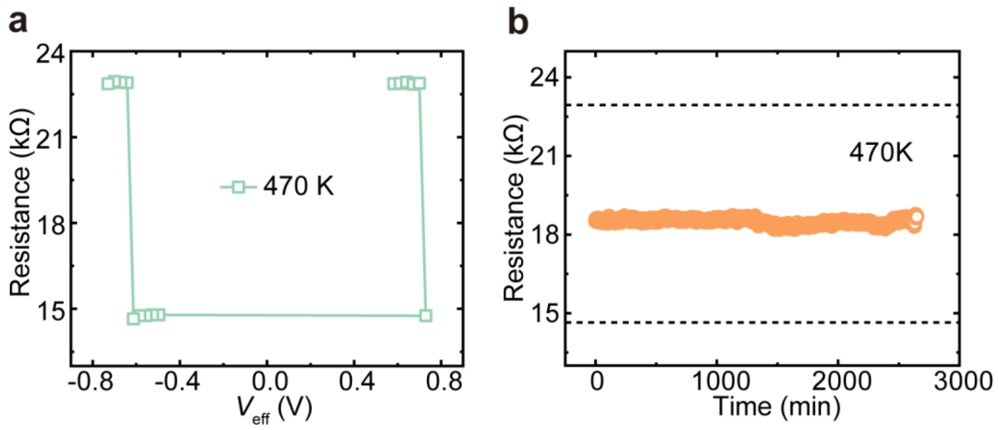

Supplementary Fig. 19 Device retention at high temperature. a *R*–*V* curves measured at 470 K. **b** Temporal evolution of the intermediate resistance state at 470 K.

Supplementary Note 8. Magnetic-Field Robustness of Devices in Different Directions

To evaluate the magnetic-field robustness of the device, external magnetic fields were applied and subsequently removed, and the resistance state was monitored at zero field[2]. A perpendicular magnetic field of up to 2 T was applied to the device and then removed, as shown in Fig. 2c in the main text. Although the device resistance varies during field application due to the canting of the free and reference layer magnetizations toward the field direction, the resistance

always returns to the same initial value once the field is removed, independent of the maximum applied field amplitude.

This field-removal invariance originates from the antiferromagnetic IrMn layer, whose exchange bias is insensitive to external magnetic fields and remains pinned along the in-plane direction. After the external field is removed, the free and reference layers relax back to their original configurations under the combined effect of the IrMn exchange bias and the SAF RKKY coupling, thereby restoring the original resistance state[7–9].

In addition, *R*–*H* curves measured under magnetic fields applied along the x and y directions up to 500 mT (well above the saturation field), together with the corresponding field-robustness data, are provided in Supplementary Fig. 20. In all cases, the zero-field resistance state is fully recovered after field removal, further confirming the strong magnetic-field robustness of the device.

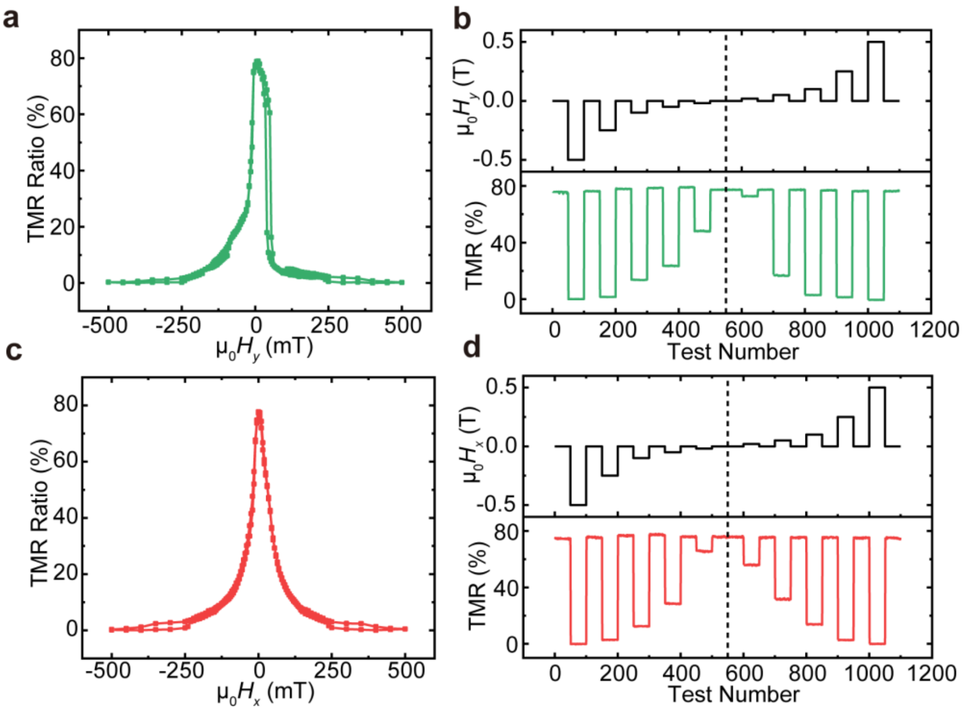

Supplementary Fig. 20 Magnetoresistance characterization of the device. a *R*–*H* curve under large magnetic field applied along the y direction. **b** Resistance variation when ±y magnetic fields are applied and removed, showing the device's resilience to y-direction fields. **c** *R*–*H* curve under large magnetic field applied along the x direction. **d** Resistance response to ±x magnetic fields, demonstrating the device's resilience to x-direction fields.

Supplementary Note 9. Effect of Antiferromagnetic Domains on Ferromagnetic Domains

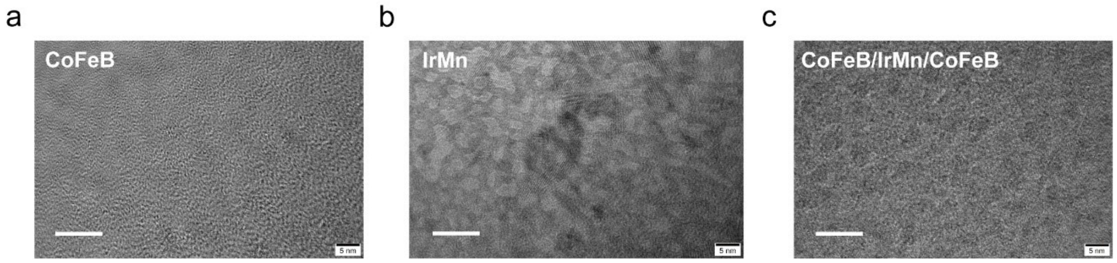

Supplementary Fig. 21 Influence of antiferromagnetic domains on ferromagnetic domain morphology. a TEM image of a 4-nm-thick $Co_{20}Fe_{60}B_{20}$ film, showing amorphous domain structures. **b** TEM image of a 6-nm-thick $Ir_{20}Mn_{80}$ film, displaying circular crystalline domains. **c** TEM image of a $Co_{20}Fe_{60}B_{20}$ (4 nm)/$Ir_{20}Mn_{80}$(6 nm)/$Co_{20}Fe_{60}B_{20}$ (4 nm) trilayer structure. The $Co_{20}Fe_{60}B_{20}$ layer exhibits circular crystalline domains, indicating morphological modulation by the adjacent $Ir_{20}Mn_{80}$ layer. Scale bar: 10 nm.

We performed TEM characterization on three types of thin-film structures: 4 nm $Co_{20}Fe_{60}B_{20}$ (hereafter referred to as CoFeB), 6 nm $Ir_{20}Mn_{80}$ (IrMn), and a trilayer structure of CoFeB (4 nm)/IrMn (6 nm)/CoFeB (4 nm). The surface domain morphologies obtained from these measurements are shown in Supplementary Fig. 21. The CoFeB single layer exhibits an amorphous domain structure, whereas the IrMn layer displays circular crystalline grains with an average grain diameter of ~7.71 nm (see main text for statistical analysis). In contrast, the top CoFeB layer in the CoFeB/IrMn/CoFeB trilayer shows a distinct transformation in morphology, exhibiting circular crystalline grains with an average diameter of ~8.47 nm. This indicates that the domain structure of CoFeB in the trilayer is influenced by the underlying IrMn layer, suggesting interfacial coupling that induces a morphological transition from an amorphous to a more ordered grain structure.

Supplementary Note 10. BCM neuron network implementation

Owing to the thermally assisted nature of the switching process, our devices are not limited to conventional pair-based STDP, but can also support more general learning rules. Here, we demonstrate how the BCM learning rule can be implemented using our devices. The BCM model describes long-term synaptic weight evolution governed by both the stimulus frequency and a history-dependent adaptive threshold.

Triplet-STDP (considered to capture second-order effects)

In practice, the BCM rule can be effectively realized using a triplet-based STDP formulation in which the weight update depends not only on spike pairs but also on triplets through additional state traces (capturing the "history dependence" introduced by thermal accumulation), As shown in Supplementary Fig. 22. Using the standard trace-based formulation, the update at a postsynaptic spike and a presynaptic spike can be written as[17]

$$\Delta W = \begin{cases} \Delta W^{+} = \mathrm{e}^{\frac{-\Delta t}{\tau_{+}}}\left(A_2^{+} + A_3^{+}\mathrm{e}^{\frac{-\Delta t_1}{\tau_{\mathrm{y}}}}\right) \\ \Delta W^{-} = -\mathrm{e}^{\frac{\Delta t}{\tau_{-}}}\left(A_2^{-} + A_3^{-}\mathrm{e}^{\frac{-\Delta t_2}{\tau_{\mathrm{x}}}}\right) \end{cases} \tag{9}$$

where ΔW denotes the weight change of an EB-MTJ synapse induced by spike interactions.

$\Delta W = \Delta W^{+}$ evaluated at $t = t_{\text{post}}$ corresponds to the post–pre–post triplet case (LTP), whereas $\Delta W = \Delta W^{-}$evaluated at $t = t_{\text{pre}}$ corresponds to the pre–post–pre triplet case (LTD). $\Delta t = t_{\text{post}} - t_{\text{pre}}$ is the time interval between the presynaptic spike and the postsynaptic spike. t'_{pre} and t'_{post} denote the time instants of the previous presynaptic spike and the previous postsynaptic spike in the pre–post–pre or post–pre–post triplet sequence, respectively. Accordingly, $\Delta t_1 = t'_{\text{post}} - t_{\text{post}}$ is the interval between two consecutive postsynaptic spikes, and $\Delta t_2 = t'_{\text{pre}} - t_{\text{pre}}$ is the interval between two consecutive presynaptic spikes.

Parameters A_2^+, A_2^-, A_3^+, and A_3^- are the amplitude coefficients of pair-based and triplet-based potentiation/depression terms, respectively, while $\tau_+$and $\tau_-$are the characteristic time constants for the pair timing dependence. τ_{x} and τ_{y} are the time constants governing the decay of the additional triplet “trace” terms associated with presynaptic and postsynaptic history, respectively[17].

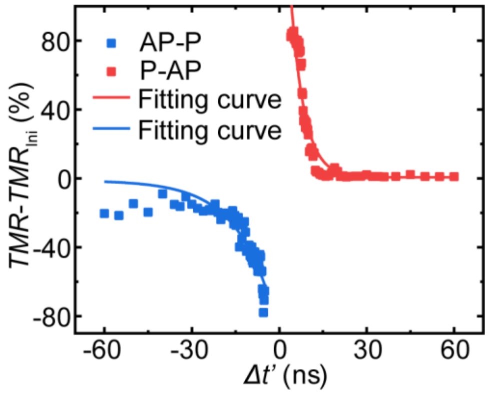

Supplementary Fig. 22 Triplet STDP fitting with the parameter τ_{x}, τ_{y}, A_3^+ and A_3^-

Triplet → BCM (minimal triplet rule and derived BCM threshold)

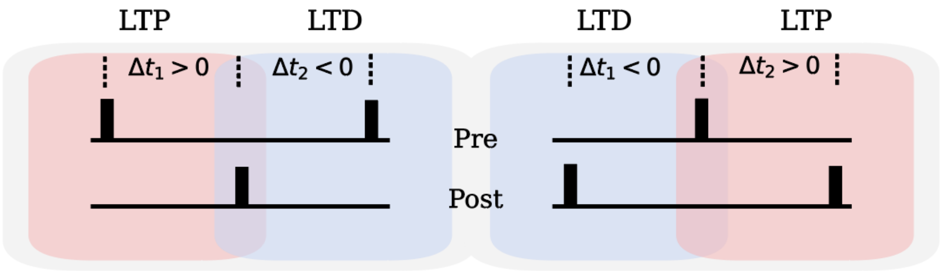

Supplementary Fig. 23 Typical triplet-pulse diagram with ‘pre–post–pre’ and ‘post–pre–post’ sequences

The triplet-STDP learning landscape can be used to generalize the BCM learning rule. Supplementary Fig. 23 illustrates the ‘pre–post–pre’ and ‘post–pre–post’ sequences used in the triplet protocol. The mathematical model of BCM learning can be expressed as[17,18]

$$\frac{\mathrm{d}W}{\mathrm{d}t} = \rho_{\text{pre}}\, \phi(\rho_{\text{post}}, \theta), \tag{10}$$

where ρ_{pre} and ρ_{post} are the frequencies of presynaptic and postsynaptic spikes, respectively, and $\phi(\rho_{\text{post}}, \theta)$ is a scalar function of ρ_{post} with a threshold frequency θ. Here, we define $\rho_{\text{pre}} = 1/\Delta t_1$ and $\rho_{\text{post}} = 1/\Delta t_2$. Synaptic depression ($\Delta W < 0$) occurs if $\phi(\rho_{\text{post}} < \theta, \theta) < 0$, whereas synaptic potentiation ($\Delta W > 0$) occurs if $\phi(\rho_{\text{post}} > \theta, \theta) > 0$; no synaptic change

occurs at the modification threshold where $\phi(\rho_{\mathrm{post}}, \theta) = 0$.

By relating triplet-STDP to the BCM rule, Eq. can be further expressed as[18,19]

$$\frac{\mathrm{d}W}{\mathrm{d}t} = -A_2^- \tau_- \rho_{\mathrm{pre}}\rho_{\mathrm{post}} - A_3^- \tau_- \tau_{\mathrm{x}} \rho_{\mathrm{pre}}^2\rho_{\mathrm{post}} + A_2^+ \tau_+ \rho_{\mathrm{pre}}\rho_{\mathrm{post}} + A_3^+ \tau_+ \tau_{\mathrm{y}} \rho_{\mathrm{pre}}\rho_{\mathrm{post}}^2, \quad (11)$$

where $A_2^{\pm}$ and $A_3^{\pm}$ are the fitted amplitudes of the pair-based and triplet-based potentiation/depression components, respectively, and τ_+, τ_-, τ_{x}, and $\tau_{\mathrm{y}}$are the corresponding time constants extracted from device-fitted triplet-STDP curves.

For the minimal triplet model (used to obtain an explicit BCM-like threshold), we set A_2^+ and A_3^- to 0 and the dominant LTD and LTP contributions reduce Eq. to a BCM-form rate dependence:

$$\frac{\mathrm{d}W}{\mathrm{d}t} = \rho_{\mathrm{pre}}\rho_{\mathrm{post}}(-A_2^- \tau_- + A_3^+ \tau_+ \tau_{\mathrm{y}} \rho_{\mathrm{post}}) \propto \rho_{\mathrm{pre}}\rho_{\mathrm{post}}(\rho_{\mathrm{post}} - \theta), \quad (12)$$

with the corresponding modification threshold

$$\theta = \frac{A_2^- \tau_-}{A_3^+ \tau_+ \tau_{\mathrm{y}}}. \quad (13)$$

Using our experimentally fitted parameters, we obtain

$$\theta \approx \frac{2.7\times 0.787\ \mathrm{ns}}{322\times 0.789\ \mathrm{ns}\times 13\ \mathrm{ns}} \approx 6.43\times 10^5\ \mathrm{Hz} \approx 0.643\ \mathrm{MHz}. \quad (14)$$

This explicitly indicates a BCM-like transition: at low ρ_{post} the net update is depressive, whereas at sufficiently high ρ_{post} the triplet term dominates and yields potentiation, with the crossover set by θ. Final parameters extracted by the fitting curve can be concluded in the Supplementary Table S1.

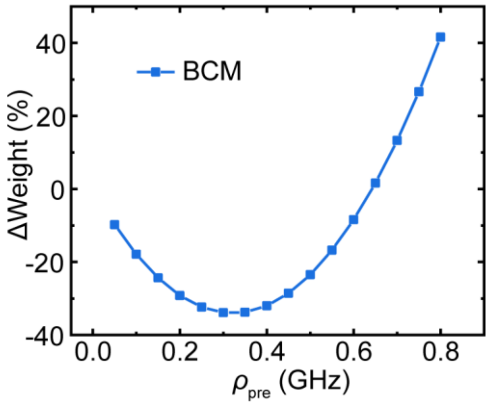

Supplementary Fig. 24 Triplet-STDP-based BCM learning rules

Supplementary Table 1 Parameters extracted from paired-STDP and triplet-STDP, optimized using the minimal triplet model.

Parameters	A_2^+	A_2^-	τ+ (ns)	τ- (ns)	A_3^+	A_3^-	τ_{x} (ns)	τ_{y} (ns)
Experimental data	3.8	2.7	0.790	0.787	322	62	4.6	13
Minimal triplet model	0	2.7	0.789	0.787	322	0	4.6	13

Network-level implementation of BCM learning

For the network implementation, we try to replace STDP with BCM training method to get the effect of BCM learning rules. Concretely, for the synaptic layers trained by local plasticity (the last fully connected layer), we record the pre- and postsynaptic spike trains over a time window of length T during each training step. From these spike trains we compute the empirical firing rates

$$\rho_{\text{pre}} = \frac{1}{TB}\sum_{t,b} s_{\text{pre}}(t,b), \rho_{\text{post}} = \frac{1}{TB}\sum_{t,b} s_{\text{post}}(t,b), \tag{15}$$

where b indexes batch samples. In the fully connected layer this yields one scalar $\rho_{\text{pre}}(\text{j})$ per input neuron and $\rho_{\text{post}}(\text{i})$ per output neuron. On top of these rates, we implement the minimal BCM form implied by equation:

$$\Delta W_{\text{ij}} = \eta\, \rho_{\text{pre,j}}\, \rho_{\text{post,i}}(\rho_{\text{post,i}} - \theta_{\text{i}})\, g(W_{\text{ij}}), \tag{16}$$

where η is an overall learning rate and $g(W) = \text{clip}(W, -1, 1)$ plays the same role as $F_{\text{pot/dep}}$ in the original pair/triplet rule (it prevents weights from drifting outside the experimentally relevant range). The sliding modification threshold θ_{i} for each postsynaptic neuron is updated as an exponential moving average of the instantaneous postsynaptic activity,

$$\theta_{\text{i}} \leftarrow \theta_{\text{i}} - \frac{\theta_{\text{i}}}{\tau_{\theta}} + \frac{\rho_{\text{post,i}}^{\text{p}}}{\tau_{\theta}}, \tag{17}$$

with $p = 2$, as in standard BCM, and τ_{θ} chosen of the same order as the slow triplet time constant τ_{y}. To ensure consistency with the device-level triplet-STDP fits, we initialize the effective BCM hyper-parameters from the experimentally extracted amplitudes and time constants. Comparing the rate-averaged expression

$$\frac{\text{d}W}{\text{d}t} = \rho_{\text{pre}}[(-A_2^{-}\tau_{-} + A_3^{+}\tau_{+}\tau_{\text{y}}\,\rho_{\text{post}})\rho_{\text{post}}] \approx \eta\, \rho_{\text{pre}}\rho_{\text{post}}(\rho_{\text{post}} - \theta), \tag{18}$$

gives $\eta \propto A_3^{+}\tau_{+}\tau_{\text{y}}$ and $\theta \propto (A_2^{-}\tau_{-})/(A_3^{+}\tau_{+}\tau_{\text{y}})$, as already stated. In the code we use these relations to set the initial scale of η and the initial value of θ, and we take $\tau_{\theta} \sim \tau_{\text{y}}$. Small dimensionless prefactors are then tuned within a narrow range to account for the discrete-time nature of training, the finite simulation window T, and numerical stability of the optimizer. We have verified that these rescalings do not change the qualitative BCM behavior: for low postsynaptic firing rates the net update is depressive, while above the fitted threshold θ the triplet term dominates and produces potentiation, as shown in Supplementary Fig. 24.

Finally, we note that in the quantization-aware training (QAT) regime used for EB-MTJ synapses, the same BCM rule is applied to the underlying continuous weights W_{real}, while the forward pass uses an effective weight

$$W_{\text{eff}} = (1 - \alpha)\, W_{\text{real}} + \alpha\, W_{\text{q}}, \tag{19}$$

where W_{q} is the 25-state device-quantized weight and $\alpha \in [0,1]$ is a mixing parameter. As $\alpha \to 1$, the BCM learning rate is smoothly reduced and eventually frozen, so that the learned weights remain consistent with the experimentally calibrated discrete conductance states. In this way, the network-level plasticity rule used in our CSNN simulations is a direct rate-based implementation of the minimal triplet to BCM mapping derived above, with parameters anchored to the device-measured pair/triplet STDP curves.

Finally, Supplementary Fig. 25 compares the test accuracy of networks trained with the STDP and BCM rules. The BCM–trained network shows a similar learning dynamics and converges to a test accuracy only ~2% lower than the STDP baseline. In this work the BCM configuration is used mainly to verify the BCM–type plasticity of our neuron–synapse model, and

its hyper-parameters were not extensively optimized. With further tuning, the BCM accuracy can be reduced to a negligible gap or even exceed that of the STDP setting.

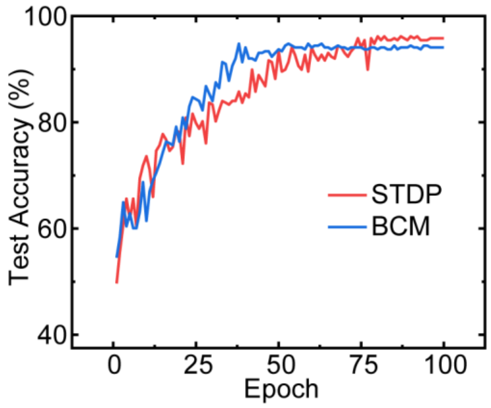

Supplementary Fig. 25 The comparison of test accuracy of STDP and BCM.

Supplementary Note 11. Neuron and synapse implementation in all-EB-MTJs CSNN

We appreciate the reviewer's request for additional detail. In our simulations, device measurements are not used only to "fit an STDP curve"; rather, multiple experimentally extracted EB-MTJ characteristics are mapped into the network model at both the synapse and neuron levels, so that network dynamics reflect thermally assisted switching physics.

Device-to-network mapping for synaptic weights (25 stable states)

First, the measured multi-level resistance states of EB-MTJs are used to store network weights. Specifically, the EB-MTJ provides 25 stable resistance states, and all synaptic weights in both convolutional layers and fully connected layers are quantized to these 25 discrete levels via a deterministic mapping $w \leftrightarrow \mathrm{TMR}$.

The TMR sequence we used for storing synaptic weight is [0, 0.0116, 0.03047, 0.08488, 0.10981, 0.12656, 0.15316, 0.19341, 0.21864, 0.23759, 0.3133, 0.37014, 0.41417, 0.41773, 0.50603, 0.53217, 0.5997, 0.63554, 0.62743, 0.62941, 0.64062, 0.66806, 0.77575, 0.85836, 0.90784].

To validate the advantage of the device's multi-level capability, we compared network training using 5-level, 15-level, and 25-level quantization (Supplementary Fig. 27); the accuracy curves show that 25-level quantization consistently outperforms lower-level quantization, demonstrating the benefit of the device's 25 stable states for weight storage.

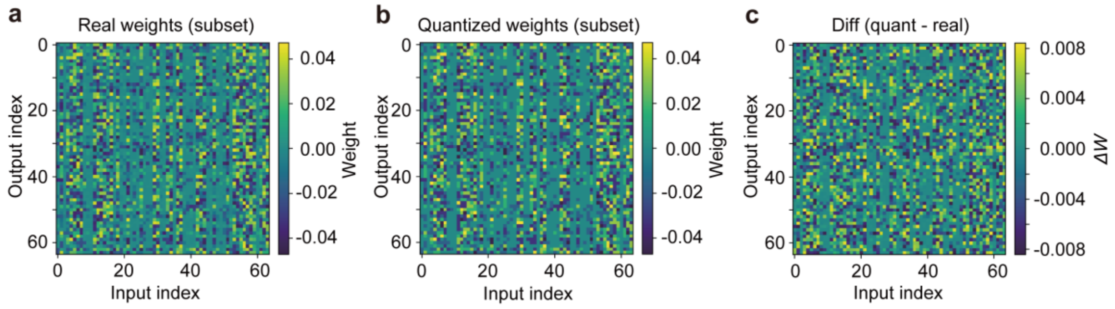

Supplementary Fig. 26 Comparison between real weights and quantized weights in our

neuron network. a,b The real weights and quantized weights in our neuron network. The input index and output index denote neuron indices in the previous and next layer, respectively; each pixel is the weight $W_{j,i}$ connecting input neuron i to output neuron j. **c** The difference (W_{quant} - W_{real}) of the real weights and quantized weights.

As shown in Supplementary Fig. 26, to stabilize training under quantization, we adopt quantization-aware training (QAT)[15], a gradual quantization schedule:

$$w_{\mathrm{eff}} = (1-\alpha)\, w_{\mathrm{real}} + \alpha\, Quant(w_{\mathrm{real}}), \tag{20}$$

where w_{real} is the real-valued weight updated by learning, $Quant(\cdot)$maps $w_{\mathrm{real}}$to the nearest of the 25 TMR levels of EB-MTJs, and α is smoothly increased from 0 to 1 during training. Thus, $\alpha = 0$ corresponds to fully real-valued training, while $\alpha = 1$ corresponds to fully device-quantized weights. This interpolation allows the network to converge stably before being fully constrained by device discreteness.

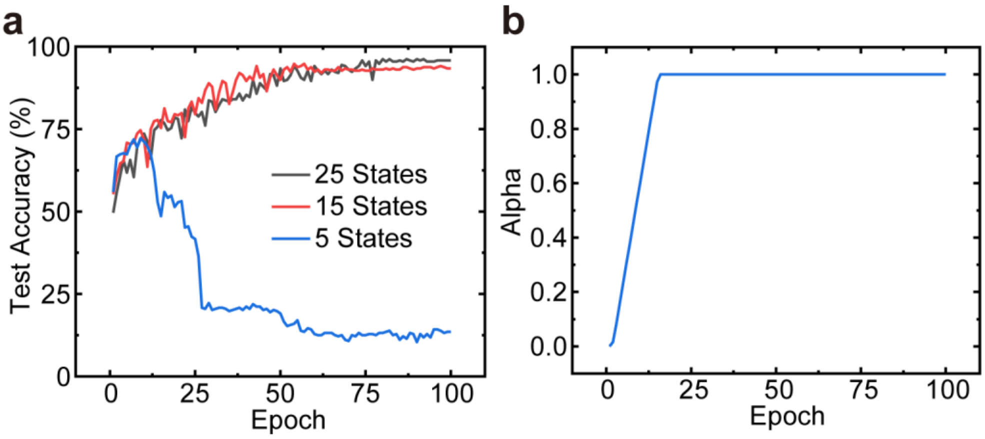

Supplementary Fig. 27 a The test accuracy of different numbers of states. **b** QAT controlled parameter α changed from 0 to 1 within 20 epoch.

Device-to-neuron mapping from thermally assisted switching dynamics (integration, leak, firing)

Second, each EB-MTJ neuron is modeled using parameters derived from measured thermally assisted dynamics, rather than assuming an abstract LIF neuron. Under input current pulses from pre-neurons, the local temperature Tevolves according to the device thermal accumulation and relaxation laws[16]:

$$T_{\mathrm{post}} = \left(\frac{J_i^2 \alpha}{K} + T_0\right)(1-\alpha) + T_{\mathrm{pre}}\alpha,\ \ \alpha = \mathrm{e}^{-\tau/\tau_0}, \tag{21}$$

and during current cessation,

$$T_{\mathrm{post}} = T_0(1-\alpha) + T_{\mathrm{pre}}\alpha, \tag{22}$$

where J_i is the input current density, Kis the combined specific heat/heat generation factor, T_0 is ambient temperature, and $\tau_0$is the device thermal response time. These equations explicitly implement integration (temperature accumulation) and leak (exponential thermal relaxation), making $T(t)$the physical analogue of membrane potential. A firing event is triggered when successive spikes raise $T(t)$above a threshold T_{th}, at which point the EB-MTJ switches state (representing threshold crossing). In the neural model, the effective leak constant and firing threshold are therefore derived from experimentally measured thermal relaxation traces and

switching-current transfer curves (e.g., extracting τ_0 and the temperature-dependent switching threshold statistics).

For the STDP training part, we employ the standard exponential pair rule:

$$\Delta w(\Delta t) = \begin{cases} A_+ \exp(-\Delta t/\tau_+) & \Delta t > 0, \\ -A_- \exp(+\Delta t/\tau_-) & \Delta t < 0, \end{cases} \tag{23}$$

where $\Delta t = t_{\text{post}} - t_{\text{pre}}$. The parameters $A_\pm$ and $\tau_\pm$ are obtained by fitting experimentally measured weight-change (or resistance-change) curves under paired pre/post stimulation protocols, as shown in Supplementary Fig. 28. In our device fitting, we obtained $\tau_+ = 3.81$ns, $\tau_- = 2.70$ns, $A_2^- = 0.787, A_2^+ = 0.789$ and incorporated them into our STDP training process.

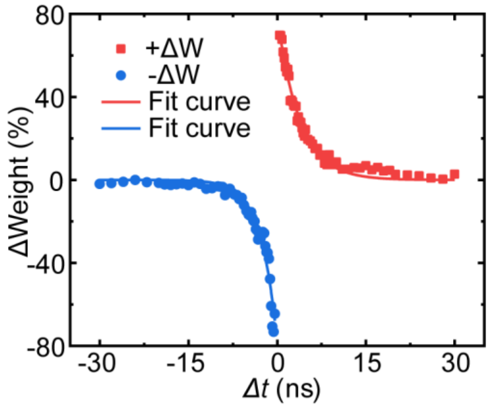

Supplementary Fig. 28 STDP rule of long-term plasticity demonstrated for paired spikes

To simplify our training process and avoid the need to record all firing times of pre and post neurons, we use the trace method to implement STDP in our neuron network. The trace-based formulation of STDP:

$$tr_{\text{pre}}[\text{i}](t) = tr_{\text{pre}}[\text{i}](t-1)\text{e}^{-\frac{1}{\tau_{\text{pre}}}} + s_{\text{i}}(t) \tag{24}$$

$$tr_{\text{post}}[\text{i}](t) = tr_{\text{post}}[\text{i}](t-1)\text{e}^{-\frac{1}{\tau_{\text{pre}}}} + s_{\text{j}}(t) \tag{25}$$

$$\Delta W_{\text{ij}}(t) = F_{\text{pot}}\left(W_{\text{ij}}(t)\right) tr_{\text{pre}}[\text{i}](t) s_{\text{j}}(t) - F_{\text{dep}}\left(W_{\text{ij}}(t)\right) tr_{\text{post}}[\text{j}](t) s_{\text{i}}(t) \tag{26}$$

where $s_{\text{i,j}}(t) \in \{0,1\}$ are spike indicators and $F_{\text{pot/dep}}$ are the bounded update functions. The chosen decay constants $\tau_{\text{pre}} = 2$ and $\tau_{\text{post}} = 4$ match the intrinsic relaxation of our EB-MTJ synapses τ_+ and τ_-.

Supplementary Note 12. Variability and Reproducibility of EB-MTJ Devices.

To investigate the variability and reproducibility of our devices, we performed a systematic statistical analysis of device uniformity from three perspectives: (i) the resistance distribution of synaptic devices with lateral sizes ranging from 80 to 160 nm, (ii) the SOT switching characteristics, and (iii) the number of accessible resistance states. The corresponding statistical results are summarized in Supplementary Figs. 29, 30, and 31, respectively.

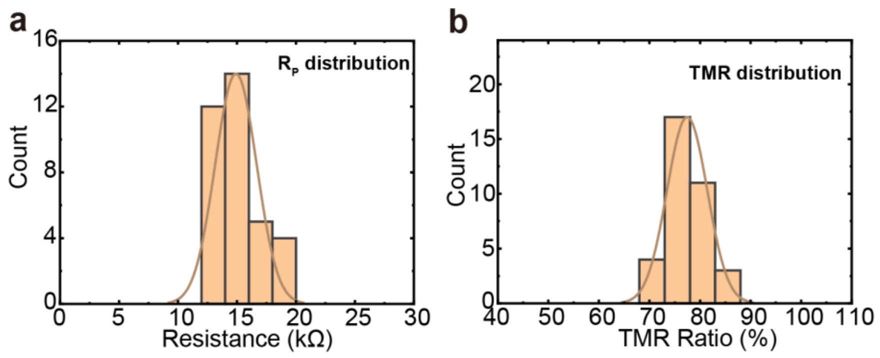

Supplementary Fig. 29 Distribution statistics of P-state resistance and TMR across multiple devices.

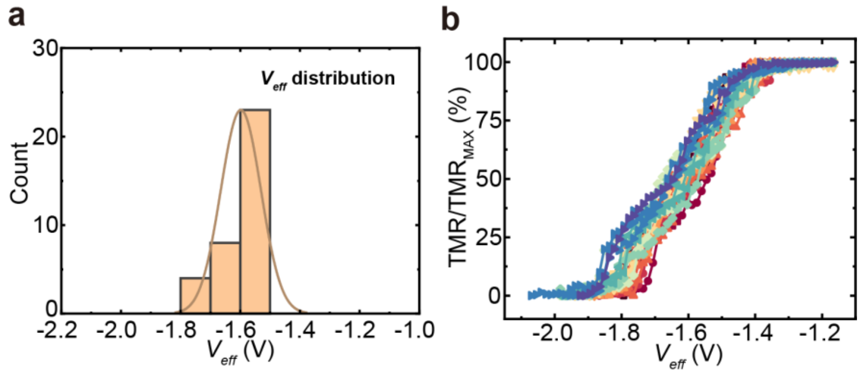

Supplementary Fig. 30 Distribution of SOT critical switching voltages and corresponding switching curves for multiple devices.

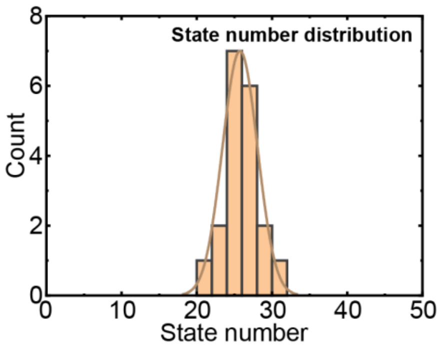

Supplementary Fig. 31 Distribution of the number of resistance states across multiple devices.

We note that, under the current laboratory-scale fabrication conditions, the P-state resistance exhibits a certain level of variation, distributed mainly in the range of 12–19 kΩ. In contrast, the TMR shows a much more concentrated distribution, primarily between 75% and 85%, indicating a relatively stable spin-transport performance despite the resistance fluctuation. Meanwhile, the SOT switching characteristics also display moderate device-to-device variation, with an approximately 10.5% difference in the threshold voltage required for complete switching into the P state. Nevertheless, all devices are able to support more than 20 stable resistance states, and the distribution of the number of states is relatively narrow, which already satisfies the basic requirements for multi-level synaptic programming in neuromorphic computing.

We attribute the observed variations mainly to the fact that these devices were fabricated as

laboratory-scale samples using manually optimized EBL exposure and etching parameters. As the process conditions are not yet fully standardized, small deviations in the actual device dimensions are unavoidable, and the etching process may also induce slight edge damage. These factors can jointly contribute to the observed spread in device resistance and TMR values[12–14].

To further verify that the observed non-uniformity mainly originates from the laboratory-scale fabrication process rather than from intrinsic material or physical limitations, we fabricated an additional batch of circular devices with a diameter of 700 nm using a commercial semiconductor foundry, while keeping the same multilayer stack. Owing to the constraints of the foundry process, the device size is larger than that of the laboratory-fabricated samples; nevertheless, this batch provides a reliable reference for evaluating process-induced uniformity.

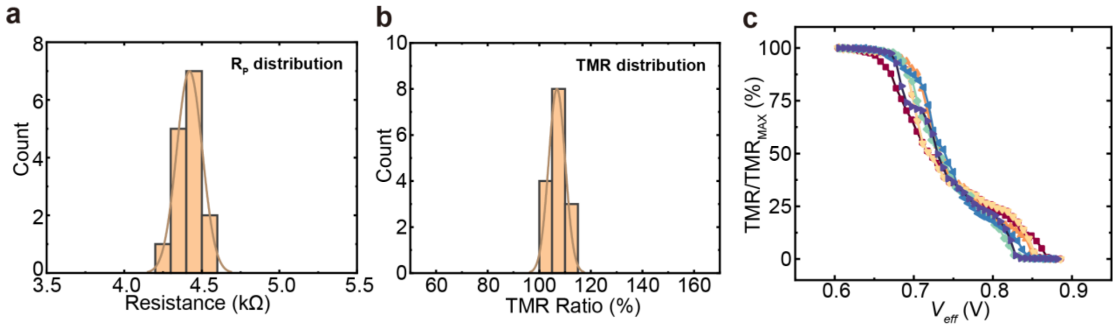

Supplementary Fig. 32 Device characteristics from foundry fabrication. a Statistical distribution of P-state resistance for multiple devices. **b** Statistical distribution of TMR for multiple devices. **c** Representative switching curves of multiple devices.

As shown in Supplementary Fig. 32, the P-state resistance of the foundry-fabricated devices exhibits a highly concentrated distribution, with the maximum-to-minimum variation being only ~7%. Meanwhile, all devices demonstrate a TMR exceeding 100%, with values mainly distributed between 102% and 112%, corresponding to a standard deviation of only 3.22%. In addition, the SOT switching curves measured under 50 ns current pulses are significantly more clustered compared with those of the laboratory-fabricated devices, further confirming the excellent device-to-device uniformity achievable under mature and well-controlled fabrication conditions.

These results strongly suggest that the uniformity of EB-MTJ synaptic devices can be substantially improved by transitioning from laboratory-scale fabrication to industrial-grade semiconductor processes. The remaining differences between the two batches are therefore not fundamental, but rather process-related, and are expected to be further mitigated through standardized lithography and etching conditions.

We further note that, although the laboratory-fabricated devices exhibit relatively larger variations, their current performance is already sufficient for the implementation of the fully EB-MTJ-based CSNN demonstrated in this work. To quantitatively assess the impact of device non-uniformity on system-level performance, we incorporated the experimentally extracted distributions of resistance states and state-to-state variations into network-level simulations.

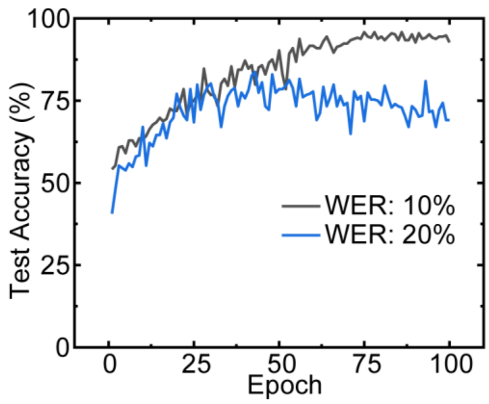

Supplementary Fig. 33 Classification accuracy as a function of the write error associated with each synaptic resistance state.

We simulate the impact of the number of synaptic resistance states on the gesture-recognition accuracy of the full EB-MTJ-based CSNN proposed in the main text, as shown in Supplementary Fig. 27a. When the number of resistance states is 15, the network achieves a maximum accuracy of 94.8%, which is only 1.4% lower than that obtained with 25 resistance states (96.2%), indicating that the network performance remains largely preserved even with a slightly reduced state number. In addition, considering that write operations based on SOT voltage pulses may inevitably introduce write errors, such as transitions to neighboring resistance states, we further analyze the influence of synaptic write error on network accuracy, as shown in Supplementary Fig. 33. Notably, even at a write error rate of 10%, the network maintains stable operation and high recognition accuracy. These results collectively demonstrate that the proposed EB-MTJ-based neuromorphic architecture exhibits strong tolerance to device non-idealities and intrinsic variations.

Finally, we would like to emphasize that the primary objective of this study is not to demonstrate large-scale device integration or state-of-the-art uniformity, but rather to introduce a monolithically integrated EB-MTJ-based CSNN architecture capable of achieving a hand-gesture recognition accuracy exceeding 96%. Importantly, the experimentally demonstrated device performance already meets the accuracy and reliability requirements of the proposed CSNN. With further improvements in device uniformity enabled by mature foundry fabrication, the system-level performance is expected to be further enhanced, rather than fundamentally limited.

Supplementary Note 13. Hysteresis loops of intermediate resistance states

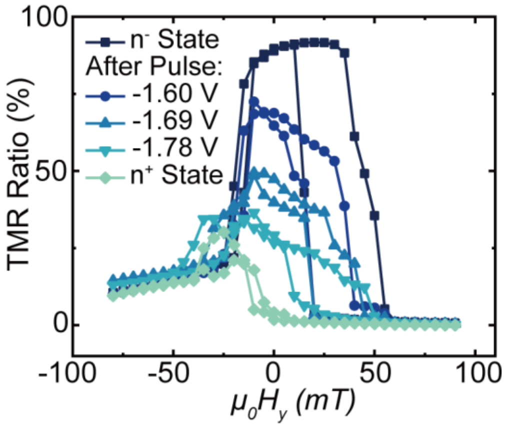

Supplementary Fig. 34 Schematic *R–H* curves of the device at different resistance states.

We measured the $R-H$ curves at different stabilized intermediate resistance states of the EB-MTJ, as shown in Supplementary Fig. 34. After programming the device into a specific intermediate resistance state, the resistance was measured as a function of the applied magnetic field, from which the effective exchange-bias field was extracted based on the loop shift. As the writing current increases, the extracted exchange-bias field continuously decreases, approaches zero, and subsequently increases in the opposite direction.

Supplementary Note 14. Temporal Stability of Intermediate Resistance States in Synaptic Devices

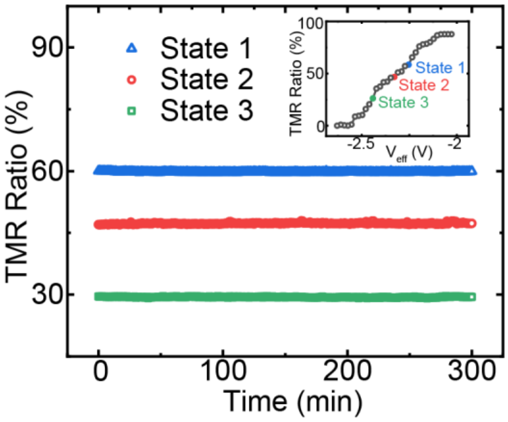

Supplementary Fig. 35 Time evolution of different intermediate resistance states in the same device. The stability of various intermediate resistance states was monitored over time in a single device.

We evaluated the retention performance of the same device under various intermediate resistance states, as shown in Supplementary Fig. 35. Owing to the exchange bias from IrMn on CoFeB, the memristive device exhibits excellent stability across all states, maintaining its resistance for over 18,000 s with fluctuations less than 2%.

Supplementary Note 15. Confusion Matrix at Best Validation Epoch

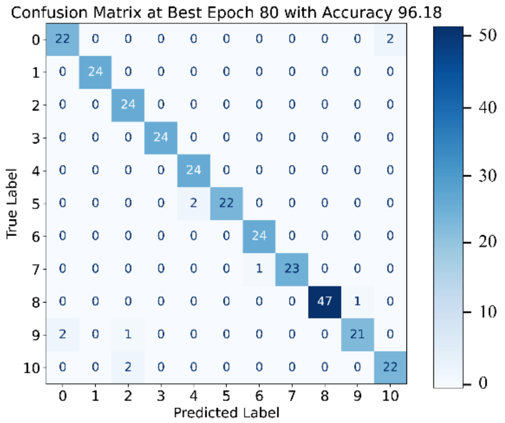

Supplementary Fig. 36 Confusion Matrix at Best Validation Epoch. Confusion matrix of the classification results at the best epoch with an accuracy of 96.18%.

Supplementary Fig. 36 depicts the confusion matrix obtained on the DVS-128 gesture dataset at the best validation epoch (epoch 80, overall accuracy = 96.18 %). Rows correspond to the true class labels (0–10) and columns to the predicted labels. Diagonal dominance confirms that the CSNN achieves near-perfect recall for most classes.

Supplementary Note 16. Trace-based modeling of STDP in EB-MTJ synaptic devices

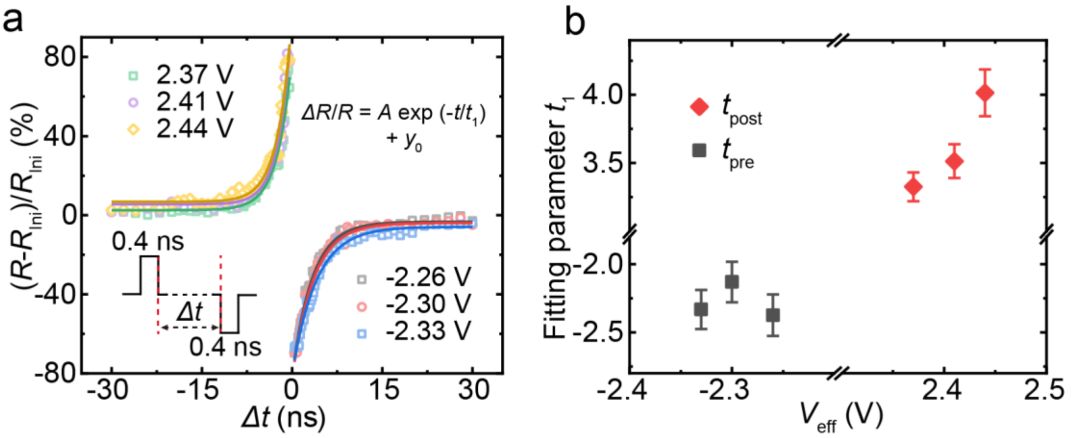

Supplementary Fig. 37 Trace-based modeling of STDP in EB-MTJ synaptic devices. a Normalised resistance $\Delta R/R$ versus interpulse delay Δt for various writing voltage, measured under a 0.4 ns SOT pulse pair (schematic inset). Solid lines are single-exponential fits. **b** Extracted t_1 values, highlighting the ranges used for τ_{pre} (black square) and τ_{post} (red rhombus) in network simulations.s

As shown in Supplementary Fig. 37, synaptic plasticity is implemented with the trace-based formulation (Eq. 28) of STDP:

$$tr_{\text{pre}}[\text{i}](t) = tr_{\text{pre}}[\text{i}](t-1)\text{e}^{-\frac{1}{\tau_{\text{pre}}}} + s_{\text{i}}(t) \qquad (27)$$

$$tr_{\mathrm{post}}[\mathrm{i}](t) = tr_{\mathrm{post}}[\mathrm{i}](t-1)\mathrm{e}^{-\frac{1}{\tau_{\mathrm{pre}}}} + s_{\mathrm{j}}(t) \quad (28)$$

$$\Delta W_{\mathrm{ij}}(t) = F_{\mathrm{pot}}\left(W_{\mathrm{ij}}(t)\right) tr_{\mathrm{pre}}[\mathrm{i}](t) s_{\mathrm{j}}(t) - F_{\mathrm{dep}}\left(W_{\mathrm{ij}}(t)\right) tr_{\mathrm{post}}[\mathrm{j}](t) s_{\mathrm{i}}(t) \quad (29)$$

where $s_{\mathrm{i,j}}(t) \in \{0,1\}$ are spike indicators and $F_{\mathrm{pot/dep}}$ are the bounded update functions. The chosen decay constants $\tau_{\mathrm{pre}} = 2$ and $\tau_{\mathrm{post}} = 4$ match the intrinsic relaxation of our EB-MTJ synapses. Device-level measurements were fitted to a single-exponential $\Delta R/R = A\exp(-t/t_1) + y_0$, across five cells the extracted t_1 averages 2.1 ± 0.2 ns for the potentiation branch and 3.4 ± 0.3 ns for depression, firmly placing τ_{pre} and τ_{post} within the experimentally accessible range.

Neuronal parameters $V_{\mathrm{rest}} = 300$ and $V_{\mathrm{th}} = 450$ were calibrated using the same pulse-train protocol that establishes LIF-like “integrate”, “fire”, and “leak” phases (Methods, main text).

Supplementary Table S2. Comparison and benchmark among different typical multi-state synapse devices

Category	SOT-MLC[20]	DW-MTJ[21]	DW-MTJ[22]	Chiral DW-MTJ[23]	RS-MTJ[24]	SOC DW-MTJ[25]	Skyrmion-MTJ[26]	HfOx-RRAM[27]	**This work**
Area	1.05×4.5 μm^2	100×800 nm^2	2×50 μm^2	200×200 nm^2	200×200 nm^2	10×18.6 μm^2	120×528 nm^2	70×70 nm^2	**77×133 nm^2**
Stability	Medium	Low	Medium	Low	Low	Low	Low	Low	**High**
modulations method	Electrical	Electrical	Magnetic	Electrical	Electrical	Electrical	Electrical	Electrical	**Electrical**
Field free	Yes	No	No	No	No	Yes	Yes	Yes	**Yes**
Write time	300 ns	DC	-	200 ns	1 s	5 μs	1 ns	2.5 μs	**0.4 ns**
On-off ratio	Medium	Medium	Low	Medium	Low	Medium	Low	High	**Medium**
Endurance	High	Medium	High	Medium	Low	High	Medium	Low	**High**
Number of states	4	15-20	4	8	3	6	8	>20	**>25**
CMOS compatibility	Yes	Yes	Yes	Yes	Yes	Yes	Yes	Yes	**Yes**
Investigations method	Experiment	Experiment	Experiment	Experiment	Exp. & Simu.	Experiment	Simulation	Experiment	**Experiment**
Spiking neuron compatibility	No	No	No	No	No	No	No	No	**Yes**

Supplementary Table S3. Comparison and benchmark among different typical spiking neuron devices

Category	DW-racetrack-MTJ[28]	SAF-DW-MTJ[29]	Type-canted-MTJ[30]	Stray-field-MTJ[16]	Magnetoelectric-based[31]	DMI-skyrmion[32]	**This work**	
Neuron type	IF	LIF	LIF	LIF	LIF	LIF	**LIF**	
Area	0.25×5 μm^2	220×50 nm^2	240×80 nm^2	93.3×160.6 nm^2	45×45 nm^2	512×100 nm^2	**104×104 nm^2**	**52×52 nm^2**
Field free	No	No	Yes	Yes	Yes	Yes	**Yes**	**Yes**
Write time	50 ns	2 ns	2 ns	3 ns	1 ns	2 ns	**0.4 ns**	**0.4 ns**
On-off ratio	Medium	Low	Medium	Medium	-	-	**Medium**	**Medium**
Energy per bit	153.6 pJ	486 fJ	100 fJ	49 fJ	246 fJ	91 fJ	**190 fJ**	**56.4 fJ**
Investigations method	Experiment	Exp. & Simu.	Exp. & Simu.	Experiment	Simulation	Experiment	**Experiment**	
Synapse compatibility	No	No	No	No	No	No	**Yes**	

Supplementary References

1. Vallejo-Fernandez, G., Fernandez-Outon, L. E. & O'Grady, K. Antiferromagnetic grain volume effects in metallic polycrystalline exchange bias systems. *J. Phys. D: Appl. Phys.* **41**, 112001 (2008).
2. Du, A. *et al.* Electrical manipulation and detection of antiferromagnetism in magnetic tunnel junctions. *Nature Electronics* **6**, 425–433 (2023).
3. Cai, W. *et al.* Anatomy of Thermally Interplayed Spin-Orbit Torque Driven Antiferromagnetic Switching. Preprint at https://doi.org/10.48550/arXiv.2410.13202 (2024).
4. Schuster, C. & Fichtner, W. Parasitic modes on printed circuit boards and their effects on EMC and signal integrity. *IEEE Transactions on Electromagnetic Compatibility* **43**, 416–425 (2001).
5. Chang, C. T. M., Namordi, M. R. & White, W. A. The effect of parasitic capacitances on the circuit speed of GaAs MESFET ring oscillators. *IEEE Transactions on Electron Devices* **29**, 1805–1809 (1982).
6. Agrawal, V. *et al.* Subthreshold operation of SONOS analog memory to enable accurate low-power neural network inference. in *2022 International Electron Devices Meeting (IEDM)* 21.7.1-21.7.4 (2022). doi:10.1109/IEDM45625.2022.10019564.
7. Peng, S. *et al.* Exchange bias switching in an antiferromagnet/ferromagnet bilayer driven by spin–orbit torque. *Nat Electron* **3**, 757–764 (2020).
8. Power, S. R., Ferreira, M. S., Power, S. R. & Ferreira, M. S. Indirect Exchange and Ruderman–Kittel–Kasuya–Yosida (RKKY) Interactions in Magnetically-Doped Graphene. *Crystals* **3**, 49–78 (2013).
9. Ma, M. *et al.* Optical control of RKKY coupling and perpendicular magnetic anisotropy in a synthetic antiferromagnet. *Nat Commun* **16**, 4401 (2025).
10. Krizakova, V. *et al.* Tailoring the switching efficiency of magnetic tunnel junctions by the fieldlike spin-orbit torque. *Phys. Rev. Applied* **18**, 044070 (2022).
11. Cubukcu, M. *et al.* Ultra-Fast Perpendicular Spin–Orbit Torque MRAM. *IEEE Transactions on Magnetics* **54**, 1–4 (2018).
12. Zhao, W. *et al.* Failure Analysis in Magnetic Tunnel Junction Nanopillar with Interfacial Perpendicular Magnetic Anisotropy. *Materials* **9**, (2016).
13. Ziaur Rahaman, Sk. *et al.* Process-induced magnetic tunnel junction damage and its recovery for the development of spin–orbit torque magnetic random access memory. *Journal of Magnetism and Magnetic Materials* **565**, 170296 (2023).
14. Zhang, L., Zhou, J., Li, H., Shen, L. & Feng, Y. P. Recent progress and challenges in magnetic tunnel junctions with 2D materials for spintronic applications. *Appl. Phys. Rev.* **8**, 021308 (2021).
15. Krestinskaya, O., Zhang, L. & Salama, K. N. Towards Efficient RRAM-based Quantized Neural Networks Hardware: State-of-the-art and Open Issues. in *2022 IEEE 22nd International Conference on Nanotechnology (NANO)* 465–468 (2022). doi:10.1109/NANO54668.2022.9928590.
16. Zhu, D. *et al.* Thermally Driven Leaky-Integrate-and-Fire Spintronic Neurons with Stray-Field-Enabled Self-Reset for Neuromorphic Computing. *IEEE Electron Device Letters* 1–1 (2025) doi:10.1109/LED.2025.3580276.
17. Wang, Z. *et al.* Toward a generalized Bienenstock-Cooper-Munro rule for spatiotemporal

learning via triplet-STDP in memristive devices. *Nat Commun* **11**, 1510 (2020).
18. Nie, F. *et al.* An Adaptive Solid-State Synapse with Bi-Directional Relaxation for Multimodal Recognition and Spatio-Temporal Learning. *Advanced Materials* **37**, 2412006 (2025).
19. John, R. A. *et al.* Ionic-electronic halide perovskite memdiodes enabling neuromorphic computing with a second-order complexity. *Science Advances* **8**, eade0072 (2022).
20. Wang, Z. *et al.* Demonstration of Spin Orbit Torque Multi-Level Cell With Enhanced State Distinction. *IEEE Electron Device Letters* **46**, 749–752 (2025).
21. Lequeux, S. *et al.* A magnetic synapse: multilevel spin-torque memristor with perpendicular anisotropy. *Sci Rep* **6**, 31510 (2016).
22. Liu, L. *et al.* Domain wall magnetic tunnel junction-based artificial synapses and neurons for all-spin neuromorphic hardware. *Nat Commun* **15**, 4534 (2024).
23. Zhang, X. *et al.* Spin-Torque Memristors Based on Perpendicular Magnetic Tunnel Junctions for Neuromorphic Computing. *Advanced Science* **8**, 2004645 (2021).
24. Krzysteczko, P., Münchenberger, J., Schäfers, M., Reiss, G. & Thomas, A. The Memristive Magnetic Tunnel Junction as a Nanoscopic Synapse-Neuron System. *Advanced Materials* **24**, 762–766 (2012).
25. Kumar, D. *et al.* Ultralow Energy Domain Wall Device for Spin-Based Neuromorphic Computing. *ACS Nano* (2023) doi:10.1021/acsnano.2c09744.
26. Huang, Y., Kang, W., Zhang, X., Zhou, Y. & Zhao, W. Magnetic skyrmion-based synaptic devices. *Nanotechnology* **28**, 08LT02 (2017).
27. Milozzi, A., Ricci, S. & Ielmini, D. Memristive tonotopic mapping with volatile resistive switching memory devices. *Nat Commun* **15**, 2812 (2024).
28. Cui, C., Liu, S., Kwon, J. & Incorvia, J. A. C. Spintronic Artificial Neurons Showing Integrate-and-Fire Behavior with Reliable Cycling Operation. *Nano Lett.* **25**, 361–367 (2025).
29. Wang, D. *et al.* Spintronic leaky-integrate-fire spiking neurons with self-reset and winner-takes-all for neuromorphic computing. *Nature Communications* **14**, 1068 (2023).
30. Wang, S. *et al.* Compact leak-integrate-fire neuron with auto-reset functionality based on a single spin–orbit torque magnetic tunnel junction device. *Appl. Phys. Lett.* **124**, (2024).
31. Jaiswal, A., Roy, S., Srinivasan, G. & Roy, K. Proposal for a Leaky-Integrate-Fire Spiking Neuron Based on Magnetoelectric Switching of Ferromagnets. *IEEE Transactions on Electron Devices* **64**, 1818–1824 (2017).
32. Dzyaloshinskii–Moriya interaction gradient driven skyrmion based energy efficient leaky integrate fire neuron. *Journal of Magnetism and Magnetic Materials* **614**, 172694 (2025).
